# CRISPR-Cas Controls Cryptic Prophages

**DOI:** 10.3390/ijms232416195

**Published:** 2022-12-19

**Authors:** Sooyeon Song, Ekaterina Semenova, Konstantin Severinov, Laura Fernández-García, Michael J. Benedik, Toshinari Maeda, Thomas K. Wood

**Affiliations:** 1 Department of Chemical Engineering, Pennsylvania State University, University Park, PA 16802, USA; 2 Department of Animal Science, Jeonbuk National University, Jeonju-Si 54896, Republic of Korea; 3 Agricultural Convergence Technology, Jeonbuk National University, Jeonju-Si 54896, Republic of Korea; 4 Waksman Institute of Microbiology, Rutgers, The State University of New Jersey, Piscataway, NJ 08854, USA; 5 Office of the Provost, Hamad bin Khalifa University, Education City, Doha P.O. Box 34110, Qatar; 6 Department of Biological Functions Engineering, Kyushu Institute of Technology, Kitakyushu 808-0196, Japan

**Keywords:** CRISPR-Cas, persisters, cryptic prophage

## Abstract

The bacterial archetypal adaptive immune system, CRISPR-Cas, is thought to be repressed in the best-studied bacterium, *Escherichia coli* K-12. We show here that the *E. coli* CRISPR-Cas system is active and serves to inhibit its nine defective (i.e., cryptic) prophages. Specifically, compared to the wild-type strain, reducing the amounts of specific interfering RNAs (crRNA) decreases growth by 40%, increases cell death by 700%, and prevents persister cell resuscitation. Similar results were obtained by inactivating CRISPR-Cas by deleting the entire 13 spacer region (CRISPR array); hence, CRISPR-Cas serves to inhibit the remaining deleterious effects of these cryptic prophages, most likely through CRISPR array-derived crRNA binding to cryptic prophage mRNA rather than through cleavage of cryptic prophage DNA, i.e., self-targeting. Consistently, four of the 13 *E. coli* spacers contain complementary regions to the mRNA sequences of seven cryptic prophages, and inactivation of CRISPR-Cas increases the level of mRNA for lysis protein YdfD of cryptic prophage Qin and lysis protein RzoD of cryptic prophage DLP-12. In addition, lysis is clearly seen via transmission electron microscopy when the whole CRISPR-Cas array is deleted, and eliminating spacer #12, which encodes crRNA with complementary regions for DLP-12 (including *rzoD*), Rac, Qin (including *ydfD*), and CP4-57 cryptic prophages, also results in growth inhibition and cell lysis. Therefore, we report the novel results that (i) CRISPR-Cas is active in *E. coli* and (ii) CRISPR-Cas is used to tame cryptic prophages, likely through RNAi, i.e., unlike with active lysogens, active CRISPR-Cas and cryptic prophages may stably co-exist.

## 1. Introduction

Along with restriction/modification [1] and toxin/antitoxin (TA) systems [2], prokaryotes utilize clustered regularly interspaced short palindromic repeats (CRISPR) and CRISPR-associated (Cas) [3] proteins to combat phages. These systems are interrelated in that some Cas proteins and TA systems have a common ancestor; for example, *Sulfolobus solfataricus* Cas2 is structurally similar to the antitoxin GhoS (an RNase) of the *Escherichia coli* GhoT/GhoS TA system [4]. In addition, in a manner similar to our discovery [2] that toxins of host TA systems inhibit phage by degrading mRNA when the phage shuts down transcription (e.g., Hok/Sok inhibits T4 phage), some Cas proteins induce host dormancy rather than degrading phage DNA to inhibit phage propagation [5]. In addition, TA systems have been found to stabilize CRISPR-Cas systems by making them addictive to the host [6].

Although CRISPR-Cas systems exclude both external lytic and temperate (lysogenic) phages [7], CRISPR-Cas systems of lysogens that target their own integrated prophages decrease long-term fitness, and either the cell dies or the prophage is lost [7,8]. In addition, the class I-E [3] CRISPR–Cas system of *E. coli* is not related to immunity for external phages [9] and appears to be inactive in the wild-type strain at standard laboratory conditions [10], due to repression by H-NS, although it is functional when induced [11]. To date, the relationship of CRISPR-Cas to cryptic prophages; i.e., those phage remnants that are unable to form lytic particles, has not been investigated.

Up to 50% of bacterial genomes may contain stably-integrated phage DNA [12], and for *E. coli*, we discovered that its nine cryptic prophages are not extraneous DNA but instead encode genes for proteins that increase resistance to sub-lethal concentrations of quinolone and β-lactam antibiotics as well as protect the cell from osmotic, oxidative, and acid stresses (by deleting 166 kb) [13]. Although these cryptic prophages do not enhance the formation of persister cells, a subpopulation of cells that weather extreme stress by entering a dormant state [14], these phage remnants facilitate the resuscitation of persisters via nutrient sensing [15]. Therefore, the bacterial cell can co-opt the genome of its former parasite to both combat stress [13] as well as revive from dormancy [15].

Given these active roles of cryptic prophages in the stress response [13] and persister cell resuscitation [15], we hypothesized here that the native *E. coli* CRISPR-Cas system plays an active role in the regulation of cryptic prophages. We find that the CRISPR-Cas system is required for inhibiting the expression of deleterious cryptic prophage genes since, if CRISPR-Cas is inactivated by preventing crRNA production, cells die due to activation of the cryptic prophage lysis proteins YdfD of Qin and RzoD of DLP-12. Hence, we discovered CRISPR-Cas is active in *E. coli* and serves to regulate its former phage foe. 

## 2. Results

**Deletions of CRISPR-Cas components reduce growth.** We assayed the importance of CRISPR-Cas in *E. coli* K-12 by testing the individual deletions of the CRISPR-Cas genes on growth in M9 glucose medium (*cas1, cas2, cas3, casA, casB, casC, casD,* and *casE*) (Figure S1) and found only the *cas2* deletion, with its kanamycin cassette insertion, had an effect: *cas2* causes 40% slower growth in rich medium compared to the wild-type strain (specific growth rate of 0.79 ± 0.21/h vs. 1.3 ± 0.11/h, respectively). Similarly, in minimal glucose (0.4 wt%) medium, deletion of *cas2* also reduces growth by 33% (0.42 ± 0.02/h vs. 0.62 ± 0.02/h, respectively) and reduces the yield in the stationary phase (Figure S2A). We hypothesized that the *cas2* deletion/kanamycin insertion caused a polar effect by reducing spacer production given that it is directly upstream of the spacer region [11] (Figure S3). Fittingly, inactivation of CRISPR-Cas by eliminating the CRISPR arrays in the isogenic host BW25113 (henceforth Δspacer) also reduced growth by 32% in rich medium and eliminating kanamycin resistance in the *cas2* deletion strain to prevent the polar mutation affecting the spacer region restored nearly wild-type growth (Figure S2A) as well as reduced toxicity (Figure S2B) relative to the *cas2* Ωkan^R^ strain. Therefore, the *E. coli* CRISPR-Cas represses some processes that inhibit growth via crRNA and, by this criterion, is active, providing a clear advantage to the host.

**CRISPR-Cas increases single-cell resuscitation.** Since deletions of CRISPR-Cas components decrease growth, we tested for their effect on persister cell resuscitation using single-cell microscopy. Persister cell resuscitation is germane in that the dormant cells are highly stressed and have limited resources for their revival via activation of hibernating ribosomes [16,17,18]; for example, we have shown that inhibiting ATP synthesis leads to a 5000-fold increase in persister cell formation [19]. Since we discovered a facile means for converting the whole population of cells into persister cells [16,17,19], which has been used by at least 17 independent labs to date with various bacterial species [20], these stressed cells are an excellent model for testing the effects of CRISPR-Cas on *E. coli* physiology. 

**Figure 1:**
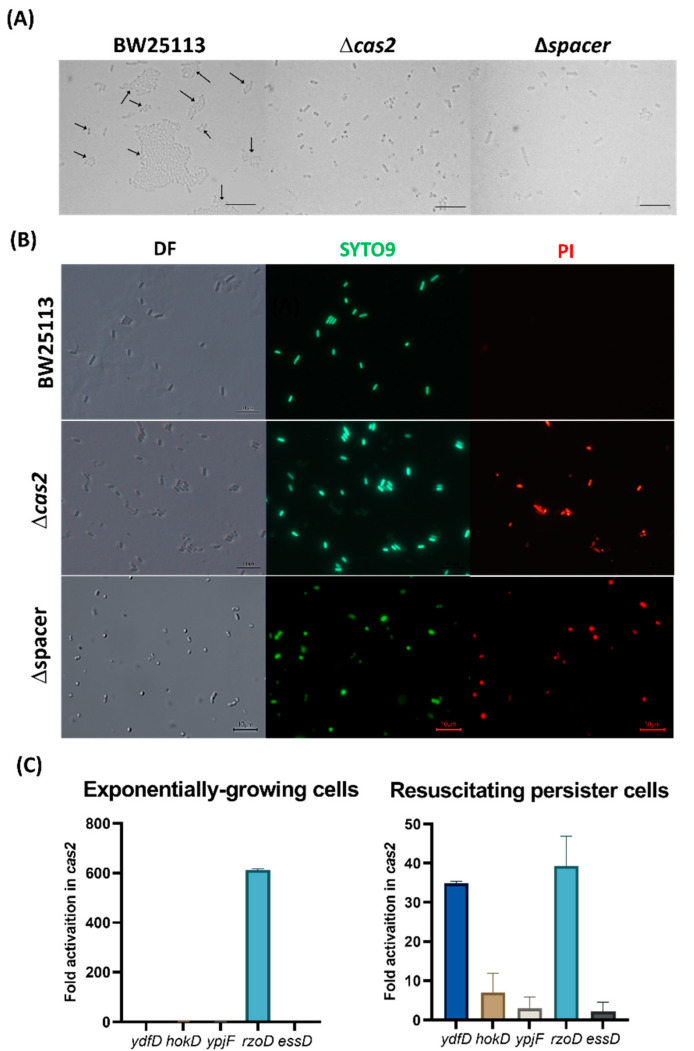
Inactivating CRISPR-Cas eliminates persister cell resuscitation by activating cryptic prophage lytic proteins, causing cell death. (**A**) Single-cell persister resuscitation for wild-type BW25113, the *cas2* mutant*,* and the Δspacer mutant after 6 hours on 0.4 wt% glucose minimal medium. Black arrows indicate cells that resuscitate, and the scale bar indicates 10 µm. Cells were observed using light microscopy (Zeiss Axioscope.A1). Representative results from two independent cultures are shown, and tabulated cell numbers are in Table S1. (**B**) LIVE/DEAD staining of resuscitating persister cells shows the *cas2* and Δspacer mutations cause cell death. DF is dark field, SYTO9 is a membrane permeable stain for nucleic acids (green), and PI is propidium iodide, which is a membrane impermeable stain for the nucleic acids of dead cells (red). Representative results from two independent cultures are shown, and tabulated cell numbers are in Table S2. (**C**) The *cas2* mutation derepresses the cryptic prophage lysis genes *ydfD* (in resuscitating persister cells) and *rzoD* (in both resuscitating persister cells and exponentially-growing cells). Five lytic genes from three cryptic prophages were checked by qRT-PCR: *ydfD* (Qin), *hokD* (Qin), *ypjF* (CP4-57), *essD* (DLP-12), and *rzoD* (DLP-12). Error bars indicate standard deviations from two independent cultures.

Here, we found that deletion of *cas2* reduces persister cell resuscitation by 31-fold (Figure 1A, Table S1). Persister cell resuscitation was similarly affected (15-fold reduction) in the Δspacer strain (Figure 1A, Table S1). Since the *cas2* mutant grows more slowly than the wild-type in rich medium, as a control, we tested an *E. coli* mutant that grows 22% slower than the wild-type, *ssrA*, to confirm that the slower growth does not affect persister resuscitation, and we found that the *ssrA* mutant resuscitates at nearly the same rate as the wild-type strain (Figure S4). Hence, CRISPR-Cas is active in *E. coli* and plays key roles in its growth and recovery from extreme stress.

**CRISPR-Cas prevents cell death by preventing cell lysis.** To explore how deletion of CRISPR-Cas decreases growth (including persister cell resuscitation), we checked for death among resuscitating persister cells of the *cas2* deletion strain using the Live/Dead stain. We found that inactivating CRISPR-Cas leads to a 7-fold increase in death among resuscitating cells (Figure 1B, Table S2). In addition, there were 34-fold more cells termed “ghosts” [21] that lack cytosolic material (Figure S5A), are likely dead, and have intact membranes, so these cells are not stained by the propidium iodide dye. Corroborating these results, there was 11-fold and 5-fold more death for stationary- and exponential-phase cells, respectively, when CRISPR-Cas was inactivated via *cas2* (Figure S6, Table S2). Moreover, for the Δspacer strain, there was 81-fold more cell death in the stationary-phase cells (Figure S6) and 7-fold more death for resuscitating persister cells (Figure 1B, Table S2). Critically, for the Δspacer strain, waking persister cells clearly show lysis (Figure S5A), and transmission electron microscopy images confirm this lysis with cytosolic materials seen next to lysed cells (Figure S5B). Unfortunately, this toxicity could not be reversed by CRISPR-Cas induction after placing the complete CRISPR-Cas system on the chromosome under inducible control (BW40114), since this led to more rapid growth when CRISPR-Cas was not induced as a result of the metabolic burden of this complete system; hence, inducing CRISPR-Cas reduced growth (Figure S7). These results indicate that inactivating CRISPR-Cas leads to cell death and that the mechanism for cell death is via lysis. 

**Figure 2:**
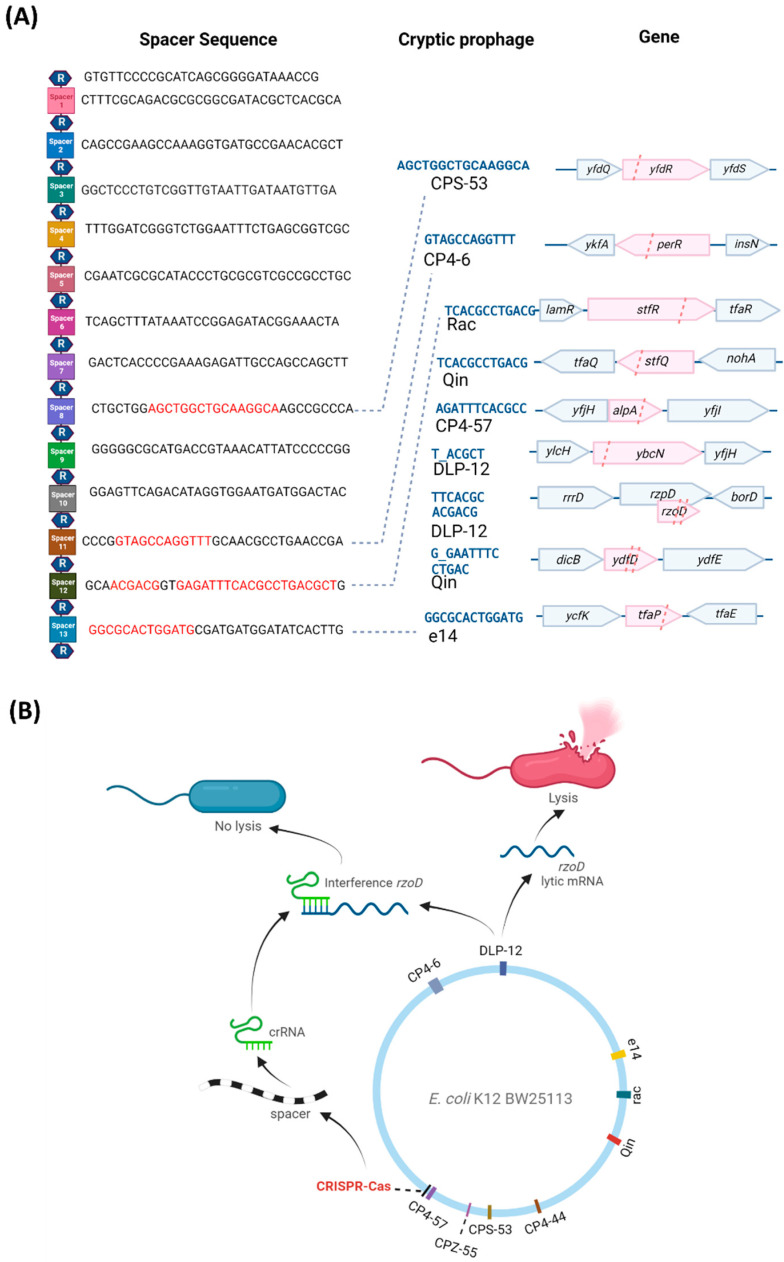
***E. coli* CRISPR-Cas spacer sequences and lytic gene inhibition mechanism.** (**A**) The 14 repeat (R, hexagon) and 13 spacer (squares) sequences of the CRISPR-Cas system (from the *iap* to *cas2* part of the *E. coli* genome) showing the cryptic prophage spacer matches (red text) and prophage DNA protospacer sequences (blue text), which include matches to seven of the nine cryptic prophages (CPS-53, CP4-6, Rac, Qin, CP4-57, DLP-12, and e14). Matches indicate mRNA binding to spacer sequences (Figure S8). Pink highlights and pink dashed lines indicate the spacer positions relative to the cryptic prophage genes. (**B**) A schematic for a hypothetical mechanism by which CRISPR-Cas controls cryptic phage lytic genes.

**CRISPR-Cas reduces cryptic prophage lysis gene mRNA.** We hypothesized that since CRISPR-Cas systems inhibit some phage before they become lysogens [7,8], the *E. coli* system may be preventing cell death by repressing certain cryptic prophage genes. To test this hypothesis, we first examined the *E. coli* CRISPR-Cas system for spacers related to the nine cryptic prophages. *E. coli* K-12 CRISPR array located next to the *cas* operon contains 13 spacers [11,22], each containing 32 or 33 nt [23]. Between the 14 29-nt repeat sequences (5′-GTGTTCCCCGCATCAGCGGGGAfTAAACCG), we found that four of the 13 spacers contain 7 to 16 nt of perfect matches to seven of the nine cryptic prophages (DLP-12, CPS-53, CP4-6, Rac, Qin, CP4-57, and e14) (Figure 2A), based on base-pairing matches with cryptic prophage mRNA (Figure S8), indicating putative binding to cryptic prophage mRNA. Critically, spacer 12 encodes crRNA with the potential to bind mRNA from DLP-12 (*rzoD*, *ybcN*), Rac (*stfR*), Qin (*stfQ*), and CP4-57 (*alpA*) (Figure S8). In general, spacer lengths vary from 21 to 72 nt [24], with perfect complementarity of 6 to 12 nt [10]. Together, these results suggest CRISPR-Cas potentially regulates the *E. coli* cryptic prophages. Note that the second CRISPR array in *E. coli* K-12 in between genes *ygcE* and *queE* lacks spacers with sequences that match the cryptic prophages.

The presence of the cryptic prophage-related targeting spacers suggests that *E. coli* CRISPR-Cas may be preventing expression of cryptic prophage genes and, given the cell phenotype, is suggestive of prophage-encoded lethal genes; hence, we checked the mRNA levels of the cryptic prophage lysis genes encoded by these seven cryptic prophages with spacer matches. Specifically, the transcription of *ydfD* (Qin), *hokD* (Qin), *ypjF* (CP4-57), *essD* (DLP-12), and *rzoD* (DLP-12) was checked via quantitative reverse transcription polymerase chain reaction (qRT-PCR). We found that inactivation of CRISPR-Cas via the *cas2* deletion results in a 35-fold increase in *ydfD* mRNA and a 39-fold increase in *rzoD* mRNA in resuscitating persister cells (Figure 1C). RzoD is a putative DLP-12 lysis lipoprotein that we previously showed was toxic through its interaction with the toxin Hha [25] of the Hha/TomB TA system [26]. YdfD of Qin cryptic prophage has been shown to lyse cells when induced [27]. To confirm that YdfD and RzoD are toxic, we induced the production of both proteins and found they are indeed toxic (Figure S9). Moreover, deleting *cas2* to inactivate CRISPR-Cas in the same host that lacks all nine cryptic prophages (delta9) [13] has no effect on growth (Figure S10) and does not cause lysis (Figure S6). Hence, these results show CRISPR-Cas represses at least two *E. coli* cryptic prophage proteins, RzoD and YdfD, that can reduce cell growth. 

**Excision of DLP-12 is not regulated by CRISPR-Cas.** Since inactivation of CRISPR-Cas leads to derepression of the DLP-12 *rzoD* lysis gene, we checked for increased DLP-12 excision with the *cas2* deletion strain using qPCR. We found the *cas2* deletion has little impact on DLP-12 excision (Table S3). Since the DLP-12 toxin gene *essD* is not derepressed, the effect is not due to excision of the prophage. We also tested the effect of CRISPR-Cas on two other cryptic prophages with significant excision (CP4-57 and e14) [13] and found no effect of deleting *cas2* (Table S3). Hence, CRISPR-Cas does not affect cryptic prophage excision.

**CRISPR-Cas regulates lytic gene mRNA levels.** Since CRISPR-Cas does not affect cryptic prophage excision and is unlikely to degrade cryptic prophage DNA as this would be lethal [7,8], we reasoned that CRISPR-Cas, via crRNA, must be interfering with expression of the mRNA of the lytic genes to prevent production of the lytic proteins. To test this hypothesis, we investigated whether *rzoD* and *ydfD* transcript levels are increased in the *cas2* and Δspacer strains relative to the wild-type after inhibiting transcription via rifampicin. If CRISPR-Cas prevents lytic gene expression by inhibiting lytic transcripts via crRNA, then inactivating CRISPR-Cas should lead to increased *rzoD* and *ydfD* mRNA. In accordance with this hypothesis, *rzoD* mRNA was increased by 320% in the Δspacer mutant and 50% in the *cas2* mutant. Moreover, as expected, inactivating CasE increased cell lysis 49-fold compared to the wild-type strain (Figure S6, Table S2), likely by preventing CasE from properly forming crRNA from pre-crRNA; hence, there is less crRNA to inhibit the cryptic prophage mRNA from toxic genes. Similarly, preventing spacer RNA formation by inactivating Cas2 led to a 10-fold increase in CRISPR array RNA levels in resuscitating persister cells as assayed by qRT-PCR, likely due to a lack of processing of the CRISPR array into crRNA (Table S3B). Finally, deleting only spacer #12 (which encodes crRNA targeting three separate regions in the cryptic prophage mRNA of CP4-57, Rac, and Qin, Figure S8) in a markerless fashion inhibits cell growth in the stationary phase (Figure S10) due to cell lysis (Figure S6, Table S2) to roughly the same extent as deleting all 13 spacers. In contrast, deleting spacer #3 in a markerless fashion, which does not encode crRNA with matches to cryptic prophage mRNA, has little effect on overall growth (Figure S10) but does increase cell death (Figure S6, Table S2), which indicates it does serve some role. Therefore, CRISPR-Cas prevents cryptic prophage lytic gene expression likely by interfering with the transcripts from these genes (Figure 2B), specifically through spacer #12. 

## 3. Discussion

Our results reveal a new (non-canonical) role for CRISPR-Cas systems: regulation of phage fossils. The evidence for this includes that inactivating CRISPR-Cas by deleting *cas2* (i) reduces growth by 40%, (ii) nearly eliminates resuscitation from the persister state (Figure 1), (iii) causes ghost cell formation and cell death (Figure S5), (iv) increases mRNA levels of the cryptic prophage lysis genes *ydfD* and *rzoD,* and (v) increases CRISPR array levels by preventing processing into crRNA. Corroborating these results, the *cas2* deletion has no effect in a strain that lack cryptic prophage genes, and eliminating the CRISPR array with prophage-matching spacers in the wild-type strain also reduced the growth rate, eliminated persister cell resuscitation, resulted in cell death via lysis, and activated *rzoD.* Furthermore, deleting only prophage-matching spacer #12 similarly inhibited growth and caused cell lysis. Given that Cas2 is not required for CRISPR interference, it is likely that the phenotypes produced upon deleting *cas2* are the result of a polar mutation (Figure S3) that inactivates CRISPR-Cas by altering the production/processing of the spacers. 

Since there is no change in excision of DLP-12 upon inactivation of CRISPR-Cas, our results suggest the mechanism for regulating the lysis genes, *ydfD* and *rzoD,* of the cryptic prophages is via CRISPR-Cas RNA binding to the cryptic prophage mRNA (analogous to RNA interference as seen previously in procaryotic systems with CRISPR-Cas [28]) rather than cleaving excised DNA or directly cleaving the mRNA. Our proposed RNAi mechanism has precedent since a previous bioinformatics analysis of 230 phages suggested CRISPR-Cas functions in *E. coli* via endogenous gene expression rather than acting as an immune system [29]. In support of this, the excision rates of the cryptic prophages are so low that there may be insufficient excised DNA to cleave (1 excision in 10^6^ cells for CP4-57) [13], and a class I-F CRISPR-Cas system in *P. aeruginosa* uses crRNA to regulate endogenous LasR mRNA to reduce the pro-inflammatory response [30]. Moreover, *E. coli* crRNA have been shown to be activated during infection in mice [30], and the *Salmonella* spp. system is identical to the *E. coli* one (e.g., the repeats are the same), but the spacers are different, implying that it, too, is for endogenous gene expression. Critically, it has been demonstrated in vitro that the *E. coli* cascade binds ssRNA complementary to crRNA [31]. Therefore, there is ample precedent for our proposed RNA interference mechanism.

Although we identified spacer #12 sequences that probably bind and interfere with the cryptic prophages Qin 12 (including *ydfD*) and DLP-12 (including *rzoD*) mRNA, there may be another level of regulation that remains to be identified. For example, we show a spacer #12 region that would bind *alpA* mRNA in cryptic prophage CP4-57, and AlpA is a regulator that impacts persister resuscitation by sensing phosphate nutrients [15].

This new function for CRISPR-Cas acting on cryptic prophages is likely general and may explain why many species appear to have inactive CRISPR-Cas systems, as was previously thought for *E. coli* [10]; i.e., instead of protecting cells from external phages, CRISPR-Cas systems may also control resident cryptic prophages, which are prevalent. As additional evidence, we found matches in CRISPR-Cas spacers not only for *E. coli* K-12 (Figure 2A), but also for *E. coli* O157:H7, *Salmonella* spp., and *K. pneumoniae* (Figure S11). Critically, our results provide the first example where it is beneficial for the host to have an active CRISPR-Cas system that targets *inactive* integrated phages (i.e., cryptic prophages), since previous reports show targeting *active* temperate phages is deleterious, i.e., either the cell dies or the phage is lost [7,8,32]. Since *E. coli* cryptic prophages like *rac* have been present in its genome for 4.5 million years [33], the active *E. coli* K-12 CRISPR-Cas system is stable with the cryptic prophages; in fact, there has been little change in the *E. coli* spacers for at least 42,000 years [34].

Our results also indicate that, although the cryptic prophages are stable and the cell makes use of the genetic tools encoded by its former foe to combat myriad stresses [13] and to sense nutrients prior to exiting the persister state [15], the source of these tools must be elegantly regulated by CRISPR-Cas since they often harbor deleterious membrane lysis proteins like YdfD and RzoD. Similarly, host Rho has been found recently to silence cryptic prophage toxin/antitoxin systems through transcription termination [35], and H-NS silences cryptic prophages through 65 binding sites [36]. Therefore, phages may be captured by the host, but they must be tamed, and this now includes repression by CRISPR-Cas. These results also suggest a role for CRISPR-Cas in gene regulation beyond self-defense.

## 4. Materials and Methods

**Bacteria and growth conditions.** Bacteria (Table S4) were cultured routinely in lysogeny broth [37] at 37 °C, while M9 glucose (0.4 wt%) [38] was used to assay the growth rate and to resuscitate persister cells. pCA24N-based plasmids [39] were retained in overnight cultures via chloramphenicol (30 μg/mL), and kanamycin (50 μg/mL) was used for deletion mutants, where applicable. The BW25113 *cas2* and spacer deletions were confirmed by PCR (primers shown in Table S5). Spacers 3 and 12 were deleted from BW25113, and *cas2* was deleted from the strain that lacks cryptic prophages (Δ9) using the method of Datsenko and Wanner [40] using plasmid pKD4 with the primers in Table S5; kanamycin resistance was removed using pCP20 [40].

**Persister cells.** Exponentially-growing cells (turbidity of 0.8 at 600 nm) were converted nearly completely to persister cells [16,19] by adding rifampicin (100 µg/mL) for 30 min to stop transcription, centrifuging, and adding LB with ampicillin (100 µg/mL) for 3 h to lyse non-persister cells. To remove ampicillin, cells were washed twice with 0.85% NaCl and then re-suspended in 0.85% NaCl. Persister concentrations were enumerated via a drop assay [41]. 

**Single-cell persister resuscitation.** Persister cells (5 µL) were added to 1.5% agarose gel pads containing M9 glucose (0.4 wt%) medium [38], and single-cell resuscitation was visualized at 37° C via a light microscope (Zeiss Axio Scope.A1, bl_ph channel at 1000 ms exposure). For each condition, at least two independent cultures were used, with 150 to 300 individual cells assessed per culture.

**Membrane integrity assay.** To determine membrane integrity, the persister cells were analyzed with the LIVE/DEAD BacLight Bacterial Viability Kit (Molecular Probes, Inc., Eugene, OR, catalog number L7012). The fluorescence signal was analyzed via a Zeiss Axioscope.A1 using excitation at 485 nm and emission at 530 nm for green fluorescence, and using excitation at 485 nm and emission at 630 nm for red fluorescence.

**qRT-PCR.** To quantify transcription from the cryptic prophage lytic genes, RNA was isolated from persister cells that were resuscitated by adding M9 glucose (0.4 wt%) to the medium for 10 min and from exponential cells grown to a turbidity of 0.8. For quantifying transcription from the CRISPR array, RNA was isolated from persister cells that were resuscitated by adding M9 glucose (0.4 wt%) medium for 10 min. Samples were cooled rapidly using ethanol/dry ice in the presence of RNA later. RNA was isolated using the High Pure RNA Isolation Kit (Roche). The following qRT-PCR thermocycling protocol was used with the iTaq^TM^ universal SYBR^®^ Green One-Step kit (Bio-Rad, Hercules, CA, USA): 95 °C for 5 min; 40 cycles of 95 °C for 15 s, 60 °C for 1 min for two replicate reactions for each sample/primer pair. The annealing temperature was 60 °C for all primers (Table S5).

**qPCR**. To quantify prophage excision and the levels of DNA flanking the CRISPR-Cas cleavage sites, total DNA (100 ng) was isolated from exponentially-growing and persistently resuscitating cells using an UltraClean Microbial DNA Isolation Kit (Mo Bio Laboratories, San Diego, CA, USA). Excised cryptic prophage was quantified using primers for each prophage excisionase (Table S5) that only yield a PCR product upon prophage excision, and the relative amount of each target gene was determined using reference gene *purM*. The level of cryptic prophage flanking the CRISPR-Cas cleave site was quantified using primers that flank each site (Table S5). The qPCR reaction performed using CFX96 Real Time System. The reaction and analysis were conducted using the StepOne real-time PCR system (Bio-Rad).

**Transmission electron microscopy.** For transmission electron microscopy (TEM), the samples were prepared from persister cells that were resuscitated by adding M9 glucose (0.4%) medium for 10 min, then washed with 0.85% NaCl. The samples were fixed with buffer (2.5% glutaraldehyde in 0.1M cacodylate buffer, pH 7.4). The negative staining was performed with 2% uranyl acetate for 1 h, then dehydrated. The sectioned specimens were stained again with uranyl acetate and lead citrate after dehydration and embedded in resin. The TEM image was observed using a Hitachi (H-7605) instrument.

## Supplementary Materials

The following supporting information can be downloaded at: https://www.mdpi.com/article/10.3390/ijms232416195/s1. Refs. [11,13,39,40,42,43,44,45] are cited in the supplementary materials.
