# Supplementary material for: CRISPR-Cas Controls Cryptic Prophages
Source: Int J Mol Sci. 2022 Dec 19;23(24):16195. doi: 10.3390/ijms232416195 (PMC9782134; doi:10.3390/ijms232416195)
Supplement: Supplementary file 1 [file ijms-23-16195-s001.zip › ijms-2110203-supplementary.pdf]

Supporting Information

Sooyeon Song[1,2,3*], Ekaterina Semenova[4], Konstantin Severinov[4], Laura Fernández-García[1], Michael J. Benedik[5], Toshinari Maeda[6], and Thomas K. Wood[1*]

[1]Department of Chemical Engineering, Pennsylvania State University,
University Park, Pennsylvania, 16802-4400, USA
Departments of Animal Science[2] and Agricultural Convergence Technology[3], Jeonbuk National University, 587 Baekje-Daero, Deojin-Gu, Jeonju-Si, Jellabuk-Do, 54896, South Korea
[4]Waksman Institute of Microbiology, Rutgers, The State University of New Jersey, Piscataway, NJ 08854
[5]Hamad bin Khalifa University, Education City, Doha, Qatar
[6]Department of Biological Functions Engineering, Kyushu Institute of Technology, Kitakyushu, Japan 808-0196

*For correspondence. E-mail songsy@jbnu.ac.kr, tuw14@psu.edu

Tel. (+)1 814-863-4811; Fax (1) 814-865-7846

Table S1. Inactivating CRISPR-Cas eliminates persister cell resuscitation on glucose agarose gel pads. Single persister cells were observed using light microscopy (Zeiss Axio Scope.A1). The total number and waking number of persister cells are shown after 6 hours on 0.4 wt% glucose minimal medium. Fold-change in waking is relative to BW25113. These results are the combined observations from two independent experiments (independent culture results separated by "/"), and standard deviations are shown. Sample microscope images are shown in **Fig. 1.**

	**Total cells**	**Waking cells**	**% waking**	**Fold-change**
BW25113	504/382	199/248	52 ± 18	1
Δ*cas2*	516/677	6/15	1.7 ± 0.7	-30.9
Δspacer	323/174	10/7	3.6 ± 0.7	-14.6

Table S2. Inactivating CRISPR-Cas causes cell death and ghost cell formation. For resuscitated cells, persister cells were washed with PBS twice, resuscitated by M9 0.4% glucose for 10 min and stained with LIVE/DEAD reagents. Exponential cells were grown to a turbidity of 0.8 (at 600 nm) and stationary cells were grown to turbidity of 2.0. The ghost cells in the persister population were visualized using a Zeiss Axioscope.A1microscope. The results are the combined observations from two independent experiments (independent culture results separated by "/"). The microscope images are shown in **Fig. 1AB** and **Fig. S6**.

	Strains	Total cells	Dead cells	Ghost cells	% Ghost cells	fold-change ghost cells	% dead	fold-change dead cells
**Resuscitated**	**BW25113**	52/115	2/7	0/1	0.6	1	5.4	1
	**Δ*cas2***	142/106	54/38	38/13	20.6	34	37.1	6.9
	**Δspacer**	161/221	76/76	-	-	-	37.8	7.0
**Exponential**	**BW25113**	153/147	0/2	-	-	-	0.7	1
	**Δ*cas2***	59/40	½	-	-	-	3.4	4.9
	**Δspacer**	95/146	1/5				2.1	3.15
**Stationary**	**BW25113**	492/123	1/0				0.1	1
	**Δ*cas2***	1136/1736	14/18	-	-	-	1.13	11.2
	**Δspacer**	200/113	19/8				8.26	81.3
	**Δ*casE***	208/175	9/10	-	-	-	5.02	49.4
	**Δ9 Δ*cas2***	833/1541	10/13	-	-	-	1.02	
	**Δspacer3**	136/297	8/73	-	-	-	15.23	
	**Δspacer12**	513/396	213/92	-	-	-	32.38	

Table S3. CRISPR-Cas does not affect cryptic prophage excision in stationary cells but produces the CRISPR array. (**A**) Fold changes in excision are relative to the wild-type strain as determined by qPCR. (**B**) Fold changes in the CRISPR array are relative to the wild-type strain as determined by qRT-PCR (the Δspacer strain was used as a negative control). Cycle numbers (C_t) are indicated for each sample including that for the target genes as well as that of the house-keeping gene, *purM,* which was used to normalize the data. *cas2* served to inactivate crRNA production due to a polar mutation. Fold changes in the transcription of various targets (i.e., cryptic prophage CP4-57, e14, DLP-12, Spacer3, and Spacer12) with and without $cas2^+$ ($cas2^+/cas2^-$) were calculated as described earlier [42]:

$$2\text{^}{-}(C_{t\ target\_cas2+} - C_{t\ purM\_cas2+}) / 2\text{^}{-}(C_{t\ target\_cas2-} - C_{t\ purM\_cas2-})$$

(A) qPCR

**Gene**	***purM***		**CP4-57**		**e14**		**DLP-12**	
Strain	WT	*cas2*	WT	*cas2*	WT	*cas2*	WT	*cas2*
C_T	9.30 ± 0.13	9.70 ± 0.18	30.45 ± 1.73	30.37 ± 0.68	16.30 ± 0.06	17.45 ± 0.51	30.58 ± 0.49	30.05 ± 0.5
ΔC_T			21.16 ± 1.73	20.67 ± 0.70	7.00 ± 0.15	8.16 ± 0.54	21.29 ± 0.51	20.35 ± 0.53
$\Delta\Delta C_T$				-0.48 ± 0.7		1.16 ±0.54		-0.94 ±0.53
fold				1.40		-2.23		1.92

(B) qRT-PCR

**Gene**	***purM***			**Spacer3**			**Spacer12**		
Strain	WT	*cas2*	spacer	WT	*cas2*	spacer	WT	*cas2*	spacer
C_T	25.3 ± 0.5	22.2 ± 0.5	22.0 ± 0.2	30.68 ± 1.73	24.35 ± 0.93	-	33.60 ± 1.20	27.12 ± 1.22	-
ΔC_T				5.37 ± 1.08	2.19 ± 1.1	-	8.28 ± 1.31	4.96 ± 1.32	-
$\Delta\Delta C_T$					-3.20 ± 1.51	-		-3.33 ±1.86	-
fold					9.11	-∞		10.02	-∞

Table S4. *E. coli* bacterial strains and plasmids utilized.

Strains and Plasmids	Features	Source
**Strains**		
BW25113	*rrnB3* Δ*lacZ4787 hsdR514* Δ(*araBAD*)*567* Δ(*rhaBAD*)*568 rph-1*	[43]
BW25113 Δ*cas1*	Δ*cas1*, ΩKm^R	[43]
BW25113 Δ*cas2*	Δ*cas2*, ΩKm^R	[43]
BW25113 Δ*cas2* ΔKm^R	Δ*cas2*, ΔKm^R	this work
BW25113 Δ*cas3*	Δ*cas3*, ΩKm^R	[43]
BW25113 Δ*casA*	Δ*casA*, ΩKm^R	[43]
BW25113 Δ*casB*	Δ*casB*, ΩKm^R	[43]
BW25113 Δ*casC*	Δ*casC*, ΩMr·	[43]
BW25113 Δ*casD*	Δ*casD*, ΩKm^R	[43]
BW25113 Δ*casE*	Δ*casE*, ΩKm^R	[43]
BW25113 Δspacer (also called BW39292)	Δ(13 spacer region), ΩKm^R	[11]
BW40114	*placuv5::cas3 paraB8p::casABCDE12spacers*	[44]
BW25113 Δspacer3	Δ(spacer region 3), ΔKm^R	this work
BW25113 Δspacer12	Δ(spacer region 12), ΔKm^R	this work
BW40114	BW25113 *lac*UV5::*cas3 araB*8p::*casABCDE12*	[44]
BW15113 Δ9	BW15113 lacking all 9 cryptic prophages	[13]
BW15113 Δ9 *cas2*	BW15113 Δ9 *cas2,* ΔKm^R	this work
ssrA	*ssrA,* ΩKm^R	[45]
**Plasmids**		
pCA24N	Cm^R; *lacI^q*	[39]
pCA24N_*cas2*	Cm^R; *lacI^q*, $P_{T5\text{-}lac}$::*cas2*$^+$	[39]
pKD4	FRT::Kan^R::FRT, Amp^R	[40]
pCP20	FLP synthase, Amp^R	[40]

Table S5. Primers used in this study for qRT-PCR and qPCR. * indicates excision primers.

Gene	Sequence (5' to 3')
**qRT-PCR**	
purM	**F:** CGGTGTTGATATTGACGCGGG
	**R:** CAGCACGGGTTCACGATATTTTTG
*ydfD (*Qin*)*	**F:** TCAGCATTTGTGCTTGTTCTG
	**R:** CTGCCGGGATTTCGATATTA
*hokD (*Qin*)*	**F:** GCCCTGATCGTCATCTGTTT
	**R:** AGCTGTGAAGACAGCGACCT
*ypjF (*CP4-57*)*	**F:** TATGGCCTGACACTGAACGA
	**R:** GTTGTTGCCACGAAAATCCT
rzoD (DLP-12)	**F:** TGCTCTGCGTGATGATGTTG
	**R:** TCTCTCTGAGGGTGAAATAATCC
essD (DLP-12)	**F:** CTCACAGTGGGCAGCAATAG
	**R:** TATTCACCTCTCGCAGCCTT
Spacer3	**F:** GATAAACCGCTTTCGCAGAC
	**R:** AGAAATTCCAGACCCGATCC
Spacer12	**F:** AACGCCTGAACCGAGAGTT
	**R:** CTGGGAGTTCTACCGCAGAG
**qPCR**	
*CP4-57	**F:** AAGCATGTAGTACCGAGGATGTAGG
	**R:** TATGTCTCCTCACCGTCTGGTCGG
*e14	**F:** GTGCAAACATCGGTGACGAA
	**R:** TTCAGCAGCTTAGCGCCTTC
*DLP-12	**F:** CAAAAGCCATTGACTCAGCAAGG
	**R:** CGGATAAGACGGGCATAAATGA
purM 400	**F:** CTGATTGCACTCGGTTCCAG
	**R:** CGTTTTCACCGTTGGCATTG
Spacer 3-1	**F:** CATGCAATTACAACATCAGGGTA
	**R:** GCCATTGGTAAAACCTTCCA
Spacer 3-2	**F:** AAATTCCGAAAAAGCTCCTGA
	**R:** TGCTTTCAAGATTATGGCGTA
Spacer 6	**F:** CACGAAAGCCAGCCTATTCC
	**R:** CCGCTGTTTCTTTCTCCAGG
Spacer 8	**F:** GCCAGCATAATGAGATCGGC
	**R:** TTTTACCCAAACTCAGCGCG
Spacer 11	**F:** TGTGCAGTTGTACCAGTGGA
	**R:** CAACCCAGCAAAGTTTCGGA
Spacer 12-1	**F:** TGTATGTGCCCCGGTGTTAT
	**R:** GCAGATGAAGGCGCATTACA
Spacer 12-2	**F:** AGCTTTACACCTCGGCTCAT
	**R:** CCGGAACTCTTGTGTTGGTG
Spacer 12-3	**F:** TCTACAGGGAAAGGACGACC
	**R:** CTCTGCAACCAAAGTGAACCA
Spacer 13	**F:** ACAACCACTATCGCCCCTTT
	**R:**GGTAAGGCTGCATTGGGAAG
**Deletions**	
cas2	**F**: ACCTTAATGTAACATTTCCTTATTATTAAAGATCAGCTAATTCTTTGTTTGT GTAGGCTGGAGCTGCTTC
	**R**: CATTCCGCTTCCTGTTTCACTGGGAGATGCAGGCCATCGGAGTAGCTGA ACATATGAATATCCTCCTTAG
Spacer 3	**F**: GCTGGCGCGGGGAACTCGCGACCGCTCAGAAATTCCAGACCCGATCCAA AGTGTAGGCTGGAGCTGCTTC
	**R**: AAAGGTGATGCCGAACACGCTGAGTTCCCCGCGCCAGCGGGGATAAACC GCATATGAATATCCTCCTTAG

Spacer 12	**F**: CAGAGGCGGGGGAACTCCAAGTGATATCCATCATCGCATCCAGTGCGCC CGTGTAGGCTGGAGCTGCTTC	
	**R**: GGTTTGCAACGCCTGAACCGAGAGTTCCCCGCGCCAGCAGGGATAAACC GCATATGAATATCCTCCTTAG	
**Verification for *cas2* and Spacer**		
cas2	**F:** GAAAATGTACCTCCGCGCTT	
	**R:** CCATCCAAATCTACCGGGGT	
**Spacer**	**F:** GGATCGGGTCTGGAATTTCT	
	**R:** GGGGAACTCGTAGTCCATCA	

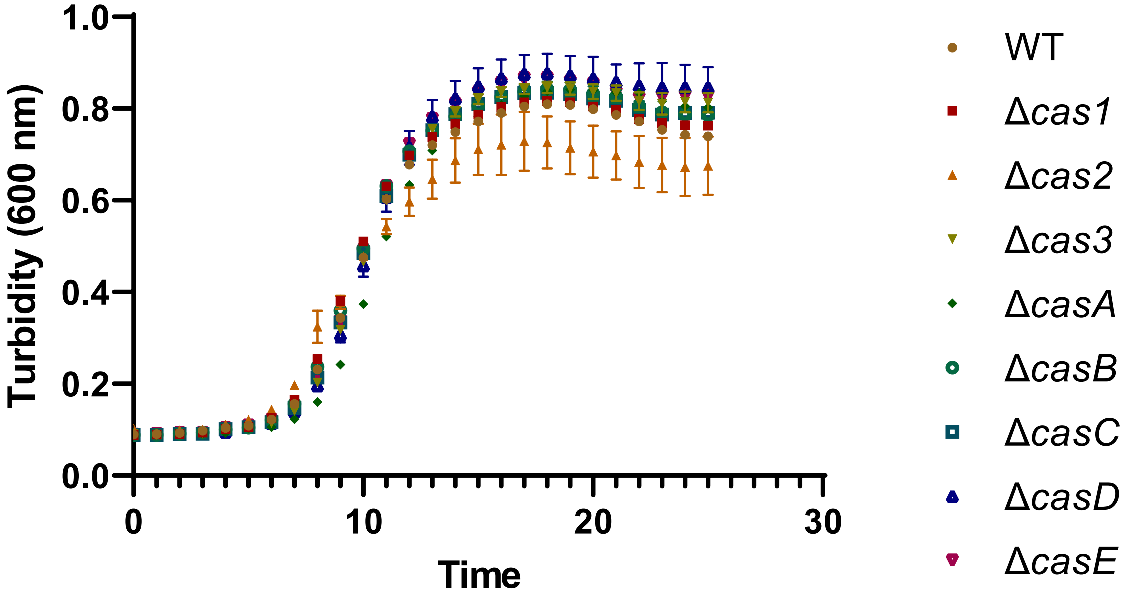

Supplementary Figure S1. Growth curves for △*cas1*, △*cas2, cas3*, △*casA*, △*casB*, △*casC,* △*casD*, and △*casE* in M9 glucose medium at 37°C. Turbidity at 600 nm shown, and error bars indicate standard deviations from two independent cultures.

(A)

(B)

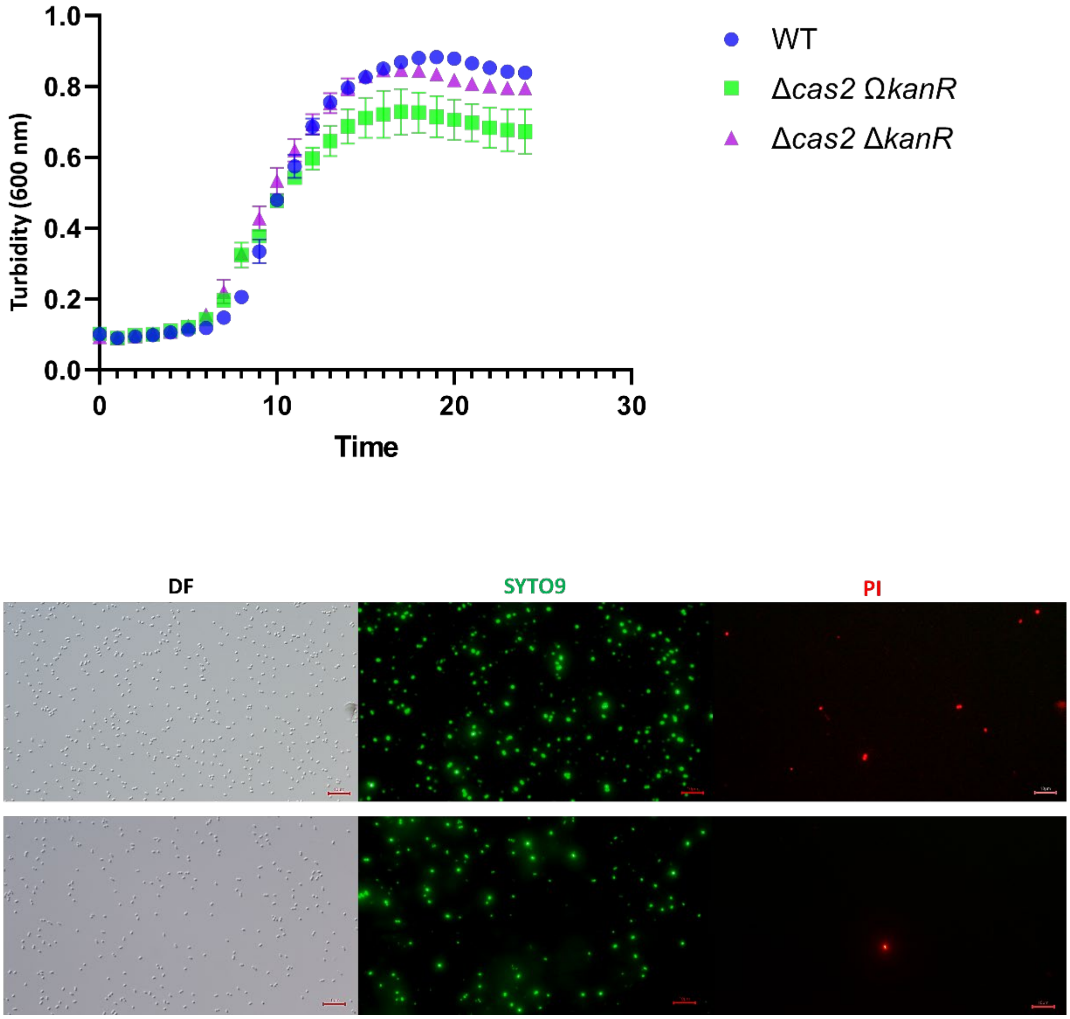

Supplementary Figure S2. Removing the kanamycin marker from the *E. coli* Δ*cas2* Ω*kan*^{R} strain to create the Δ*cas2* Δ*kan*^{R} strain **(A)** increased growth in M9 glucose medium at 37°C (turbidity at 600 nm shown and error bars indicate standard deviations from two independent cultures) and **(B)** reduced toxicity via the LIVE/DEAD assay for stationary cells showing the polar mutation due to the kanamycin marker in the Δ*cas2* Ω*kan*^{R} strain inhibits spacer formation, which results in reduced growth and increased toxicity. Representative images shown from two independent cultures.

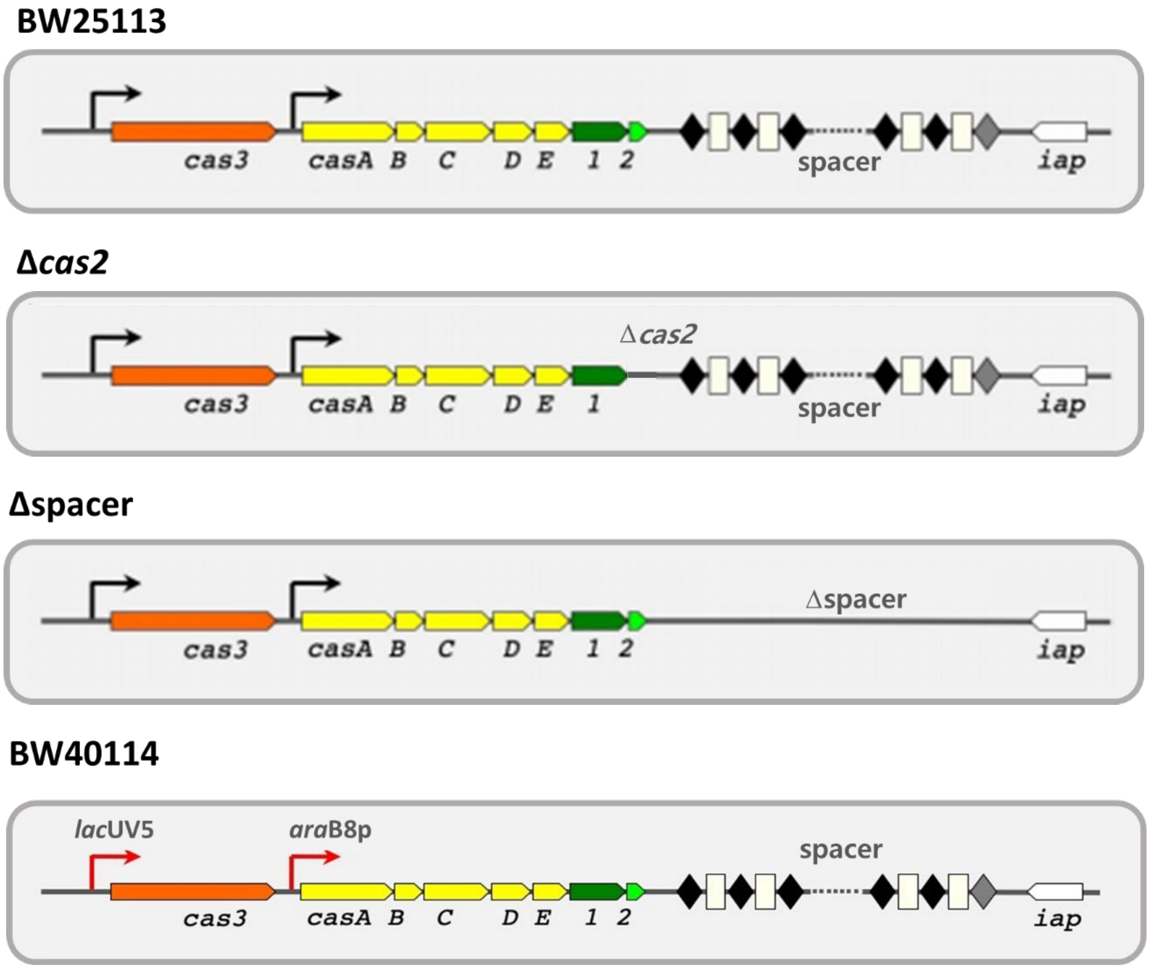

Supplementary Figure S3. Schematics of the CRISPR-Cas strains used in this study. All the strains are derivatives of isogenic host, BW25113 (see **Table S4**).

BW25113 **Δ*ssrA***

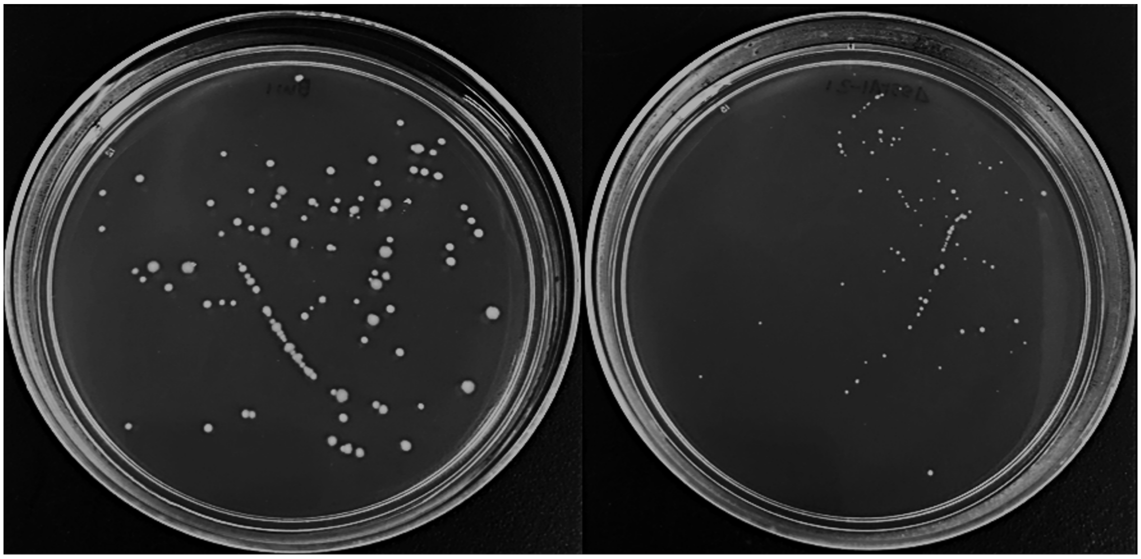

Supplementary Figure S4. As a control for growth rate and resuscitation, the *ssrA* mutation was checked and found to have no effect on persister resuscitation on LB plates although it grows 22% more slowly than the wild-type strain (1.18 ± 0.07/h vs. 1.49 ± 0.03/h, respectively). The number of *ssrA* persisters that resuscitated is 101 ± 2 vs. 100.0 ± 0.1 for the wild-type. Plates were incubated for 24 h at 37°C, and representative images shown from two independent cultures.

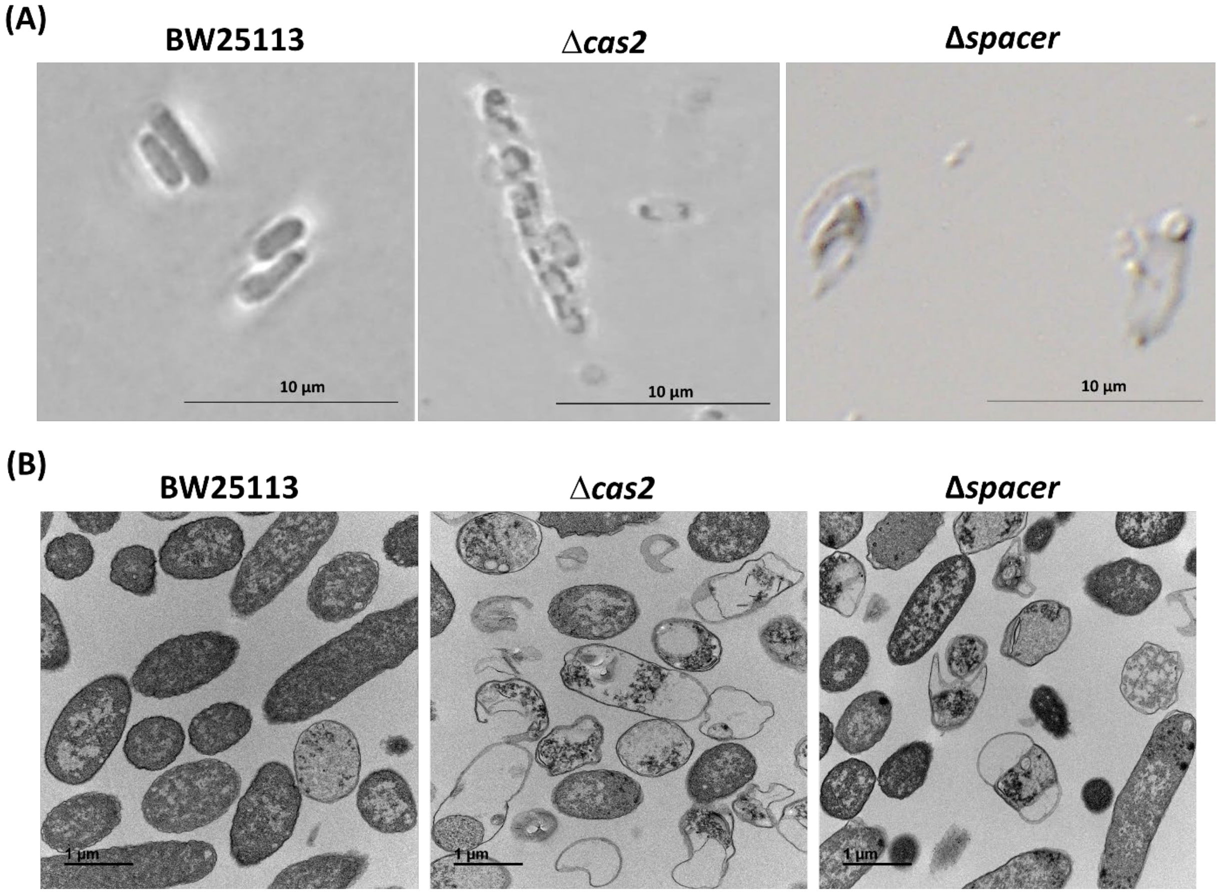

Supplementary Figure S5. CRISPR-Cas prevents cell lysis induced by cryptic phages. (**A**) Single cell persister waking of BW25113 Δ*cas2* and the Δspacer mutant on M9 0.4% glucose agar plates incubated at 37°C for 4 hours. The scale bar indicates 10µm. (**B**) TEM image for persister waking of BW25113 Δ*cas2* and the Δspacer mutant. Persister cells were resuscitated by M9 0.4% glucose for 10 min. One representative image from two independent cultures is shown. The scale bar indicates 1 µm.

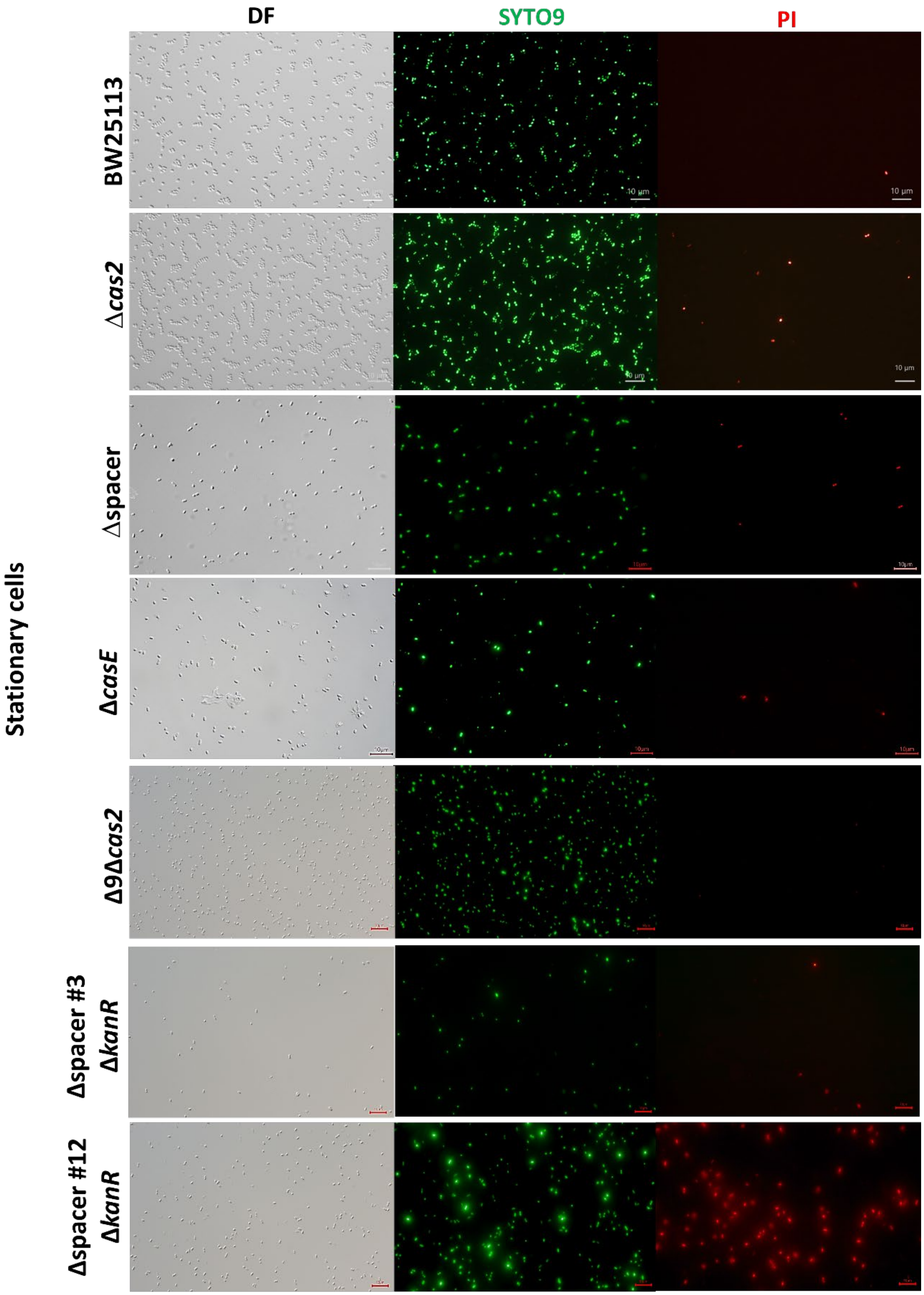
DF
SYTO9
PI
Stationary cells
BW25113
Δcas2
Δspacer
ΔcasE
Δ9Δcas2
Δspacer #3 ΔkanR
Δspacer #12 ΔkanR
10 μm

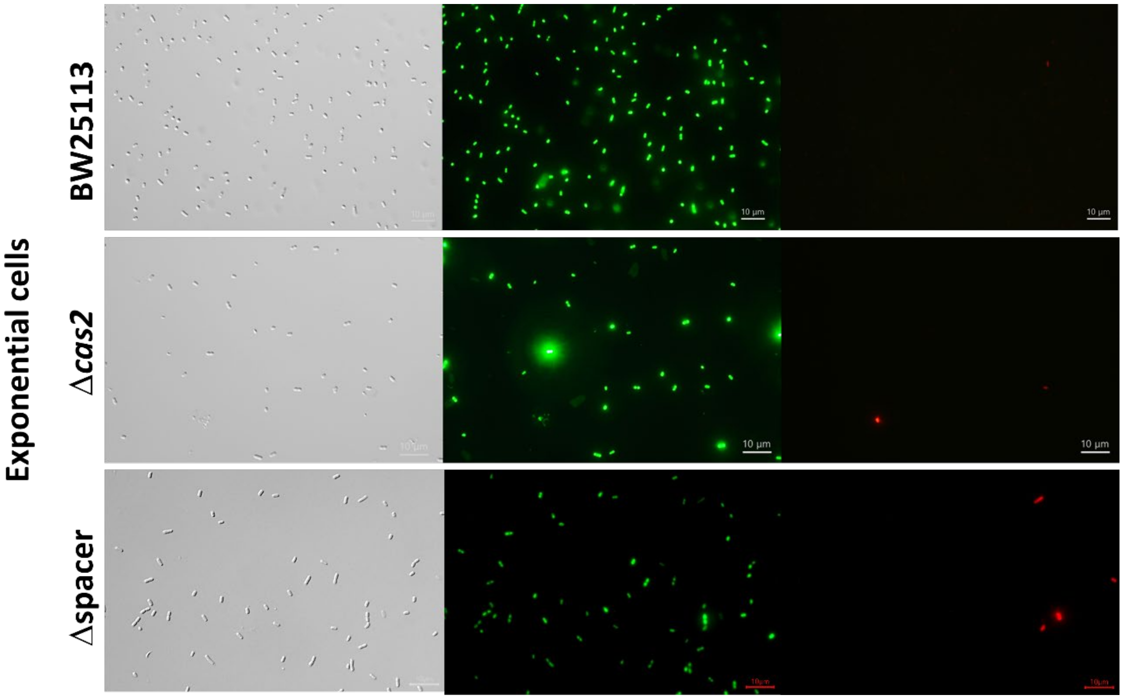

Supplementary Figure S6. LIVE/DEAD staining of stationary (turbidity 2.0) and exponential (turbidity 0.8) cells shows the *cas2*, Δspacer (all 13 spacers removed), and mutations cause cell death. DF is dark field, SYTO9 is a membrane permeable stain for nucleic acids (green), and PI is propidium iodide, which is a membrane impermeable stain for the nucleic acids of dead cells (red). Tabulated cell numbers are shown in **Table S2**, and representative images from two independent cultures shown.

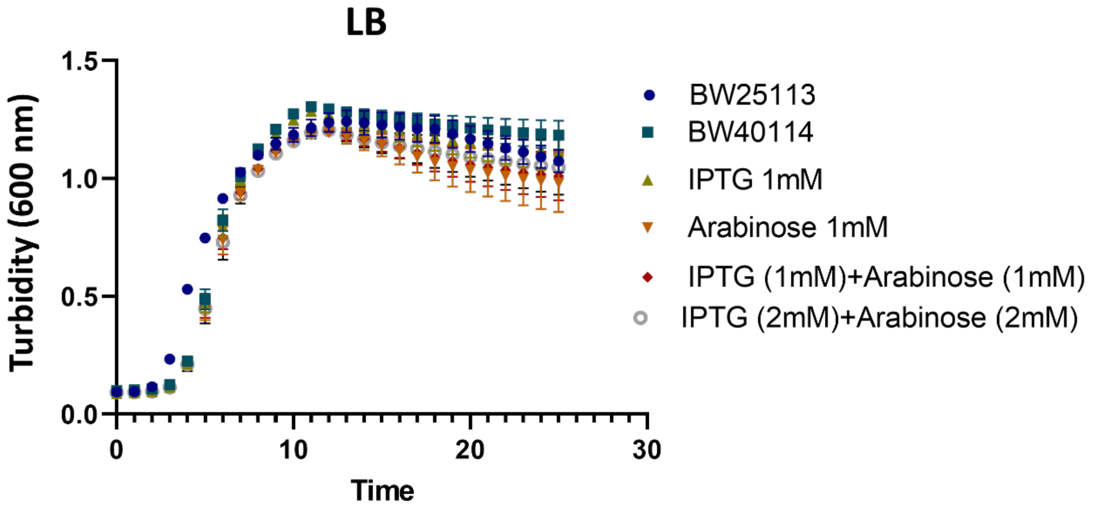

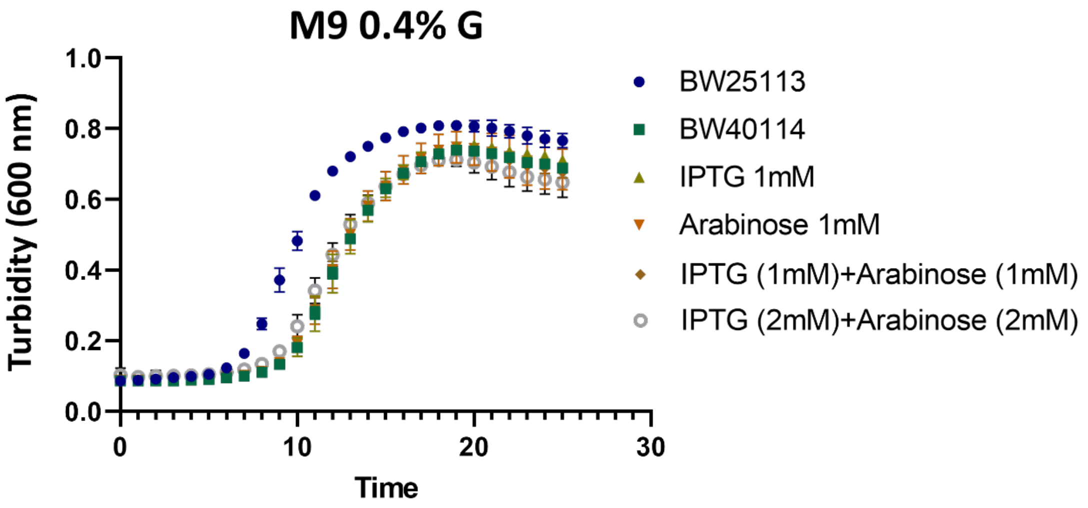

Supplementary Figure S7. Growth curves upon inducing the full CRISPR-Cas system in *E. coli* BW40114 (via IPTG and arabinose) at 37°C in LB and M9 glucose media (turbidity at 600 nm shown). The growth is reduced due to the metabolic burden of producing the complete CRISPR-Cas system. Error bars indicate standard deviations from two independent cultures.

yfdR gene (Start and stop codon)(**CPS-53,** encodes 5'-deoxynucleotidase)

```
ATGTCATTTATTAAAACTTTTTCCGGGAAGCATTTTTATTATGACAAGATAAATAAAGACGACATCGTGATTAACGA
TATCGCGGTTTCCCTTTCAAATATCTGCCGCTTTGCCGGTCATCTTTCTCACTTCTACAGTGTCGCCCAACATGCGG
                                                          Spacer 8  3'-ACCCGCCGA
TGCTTTGCAGCCAGCTGGTGCCGCAGGAATTTGCTTTTGAAGCATTAATGCATGATGCAACAGAAGCGTATTGCCAG
||||||||||||||||
ACGGAACGTCGGTCGAGGTCGTC-5'
GACATCCCCGCACCACTGAAACGCCTTCTTCCTGACTATAAACGGATGGAAGAAAAAATAGATGCAGTAATCCGTGA
GAAATACGGGTTACCTCCTGTTATGAGCACGCCAGTGAAATATGCCGATCTCATTATGCTGGCAACCGAACGCCGTG
ATCTCGGGCTTGATGATGGCTCTTTCTGGCCTGTACTGGAAGGCATCCCGGCAACAGAGATGTTCAACGTGATTCCA
CTGGCACCGGGTCATGCCTACGGGATGTTTATGGAACGTTTTAACGATTTATCGGAGTTACGCAAATGCGCATGA
```

perR gene (Start and stop codon)(**CP4-6,** encodes transcriptional regulator)

```
ATGAAGCTCTTAGCAAAAGCACCTCTAAATCTGTTACGCGCCTTTGAAGCGGCTGGTCGTACCGGAGCTTTTGCGTT
GGCCGCCTCTGAGTTGGAACTGTCACCCAGTGCGATCAGCCATGCCATCCGCAAACTGGAAAACTTGCTTGATGTAC
GCCTTTTTCAGCGCAGTACGCGAGAAATTACGCTGACGAAAGAAGGTGAAATACTGCTTGAGCACATACAGCGGGGA
TTTAACGAATTACAGCAGGGGTTGGCATTAGTGACGGCTGATGAATCCAGGCCTTTGCGCCTTCATACTGCACCGAG
TTTTGCCCATCAATGGCTTTTACCACGTCTTGGTAAGTTCATACGTGAGAACCCAAGCATCGATCTGCGACTTTCAG
CCAGCACAGAATATGCACGTTTTGAACAGGATGATTTTGATCTCGATATAGTCTACGGTGAACCCCGCCCATCACCC
TATGAGAAGATCCCGCTTGCTGTTGAAGAACTTACGCCACTGTGTTCTCCCCAACTGGCTGAGCGGCTAAAGAAACC
AGAGGATCTCTATGCGTTGACATTGATTCAGTGCGATGTGCAGTTGTACCAGTGGAAAGGATGGTTTGAGGCGAATA
AGATGACGCCACCCAATAATTATGGCCTTCGATTTGACCGTAGCTTTATGGCGATTGCTGCCGCAGTTGACGGCTTG
GGAGTTGTGCTTGAATCAAAACTACTGGCTGAACGTGAAATCGCTAGCGGTAAACTGGTATGCCCACTTGTAAACAG
TACCAGCGAAATACATTACATCGGTCATTATTTGGTTTTCCCCCAGCACCAACACATGCATTCTGCGCTTGATGTGT
                                                                 // // / / //
                                                 Spacer 11  3'-AGCCAAGTCCGCA
TCAAAACCTGGCTACTGAATGAACTCAATCTGGGTAAAATTCGTTGA
|  ||||||||||||  |
ACGTTTGGACCGATGGCCC-5'
```

stfR gene (Start and stop codon)(Rac, side tail fiber protein)

```
ATGGCAGTAAAGATTTCAGGTGTACTGAAAGACGGCACAGGAAAACCGGTACAGAACTGCACAATCCAGCTGAAAGC
AAAACGTAACAGCACCACGGTGGTGGTGAACACGCTGGCCTCAGAAAATCCGGATGAAGCCGGGCGTTACAGCATGG
ACGTTGAGTACGGTCAGTACAGCGTTATTCTGTTGGTGGAAGGATTCCCGCCGTCACATGCCGGGACCATTACCGTG
TATGAAGATTCTCAACCCGGTACGCTGAATGATTTTCTCGGTGCCATGACGGAGGATGATGCCCGTCCGGAGGCACT
GCGCCGTTTTGAACTGATGGTGGAAGAGGTGGCGCGTAACGCGTCCGCGGTGGCACAGAACACGGCAGCCGCGAAGA
AGTCAGCCAGTGATGCCAGCACATCAGCCCGTGAGGCGGCAACCCATGCGGCTGATGCTGCGGACTCAGCACGCGCA
GCCAGCACGTCAGCCGGACAGGCCGCGTCGTCGGCTCAGTCAGCGTCTTCCAGCGCAGGAACGGCATCAACAAAGGC
CACTGAAGCATCAAAAAGTGCTGCCGCTGCAGAGTCCTCAAAAAGCGCGGCGGCCACCAGTGCCGGTGCGGCGAAAA
CGTCAGAAACGAATGCTTCAGCGTCACTACAATCAGCAGCCACATCTGCATCCACCGCGACCACGAAGGCATCAGAA
GCTGCGACCTCGGCCCGGGATGCGGCGGCCTCAAAAGAAGCGGCAAAATCATCAGAAACGAACGCATCATCAAGCGC
CAGTAGTGCAGCTTCCTCGGCAACGGCGGCAGGAAATTCCGCGAAGGCGGCAAAAACGTCCGAGACGAACGCCAGGT
CTTCTGAAACGGCAGCGGGACAGAGCGCCTCGGCTGCGGCAGGCTCAAAAACAGCGGCTGCGTCGTCTGCCAGTGCA
GCGTCAACAAGTGCCGGGCAGGCCTCAGCCAGTGCCACCGCCGCCGGAAAATCGGCAGAAAGCGCCGCATCGTCTGC
TTCAACAGCCACAACGAAGGCTGGCGAAGCCACTGAACAGGCCAGCGCAGCAGCGAGGTCTGCTTCCGCAGCGAAGA
CATCCGAAACGAACGCGAAAGCGTCGGAAACAAGCGCAGAATCCTCAAAAACGGCTGCCGCATCGTCAGCCAGTTCG
GCGGCGTCATCGGCATCATCGGCGTCTGCTTCAAAAGATGAGGCGACCAGACAAGCGTCAGCAGCGAAGAGCAGCGC
CACGACGGCATCCACGAAGGCGACAGAGGCTGCTGGCAGTGCGACGGCGGCAGCTCAGAGCAAAAGTACGGCGGAAT
CCGCGGCAACGCGCGCCGAGACAGCAGCTAAACGGGCAGAGGATATTGCATCCGCCGTGGCGCTTGAGGATGCAAGT
ACGACGAAAAAGGGGATAGTACAGCTCAGCAGTGCGACCAACAGTACGTCTGAAACGCTGGCGGCAACGCCAAAGGC
AGTAAAATCAGCCTATGACAATGCAGAGAAACGTCTGCAGAAAGACCAGAACGGCGCTGATATACCCGATAAGGGAT
GCTTCCTGAACAACATTAACGCGGTCAGTAAAACAGACTTTGCTGATAAGCGTGGTATGCGTTATGTGCGGGTTAAC
GCTCCTGCAGGTGCAACATCTGGAAAATATTACCCTGTTGTTGTTATGCGTTCTGCTGGCTCAGTAAGCGAACTGGC
ATCAAGAGTCATTATCACCACGGCAACGCGAACCGCAGGCGATCCGATGAATAACTGCGAGTTTAACGGATTTGTTA
TGCCTGGTGGCTGGACTGACAGGGGGCGTTATGCTTATGGCATGTTCTGGCAATATCAAAACAATGAACGAGCCATT
CACTCAATAATGATGAGTAATAAGGGCGATGATTTGCGCTCTGTGTTCTATGTTGATGGCGCTGCTTTCCCTGTTTT
TGCGTTTATTGAAGATGGCCTGTCAATATCCGCACCTGGTGCTGATCTCGTTGTTAATGATACGACCTATAAGTTTG
GGGCAACAAATCCGGCGACTGAATGTATCGCGGCGGACGTTATCCTTGATTTTAAGAGTGGGCGTGGTTTTTATGAG
TCTCATTCGTTAATCGTTAACGATAACTTGTCGTGCAAAAAACTTTTTGCCACAGACGAAATTGTAGCGCGTGGTGG
TAATCAGATTCGAATGATAGGTGGGGAGTATGGTGCATTATGGCGTAATGATGGCGCTAAAACTTACCTGCTGCTTA
CCAATCAAGGTGATGTTTATGGTGGCTGGAATACATTAAGACCGTTTGCTATTGATAACGCAACCGGCGAACTGGTT
ATTGGAACCAAACTGTCCGCAAGTCTGAACGGTAATGCATTAACAGCAACAAAGCTGCAAACGCCAAGACGGGTTTC
TGGTGTTGAGTTTGATGGTTCCAAAGATATTACTTTAACCGCCGCGCATGTGGCTGCTTTTGCCAGAAGGGCAACGG
ATACATATGCCGATGCGGATGGTGGCGTTCCATGGAATGCCGAATCTGGCGCTTACAATGTCACCCGCTCTGGCGAC
AGCTATATTCTGGTTAACTTCTATACCGGAGTCGGAAGTTGCCGGACCCTGCAGATGAAGGCGCATTACAGAAATGG
TGGTCTGTTCTACCGTTCTTCAAGAGACGGTTATGGTTTTGAGGAAGACTGGGCAGAAGTTTATACCTCGAAAAATC
TTCCACCAGAAAGCTACCCAGTCGGCGCACCAATCCCGTGGCCATCAGATACCGTTCCGTCTGGTTATGCCCTGATG
CAGGGGCAGGCTTTTGACAAATCTGCTTACCCGAAACTTGCAGCCGCTTATCCGTCAGGCGTGATCCCTGATATGCG
                                                           ||||||||||||    || |     ||
                                             Spacer 12  3'-GTCGCAGTCCGCACTTTAGAGTGGCAGC
AACG-5'
|  ||
TGGCTGGACGATTAAGGGCAAACCTGCCAGTGGTCGGGCCGTATTGTCTCAGGAACAGGACGGCATTAAATCGCATA
CCCACAGCGCCAGCGCATCCAGTACAGATTTGGGGACGAAAACCACATCGTCGTTTGATTACGGCACTAAATCCACG
AATAACACCGGGGCACATACACACAGTGTGAGCGGCTCTACAAACTCGGCTGGAGCACACACACACTCACTAGCCAA
CGTGAACACGGCTAGTGCTAACTCCGGTGCTGGTAGTGCATCAACAAGATTGTCTGTTGTGCATAATCAAAACTATG
CAACATCATCTGCTGGCGCACATACCCACTCACTGTCCGGCACTGCTGCAAGCGCAGGTGCACACGCGCATACTGTC
GGTATTGGTGCTCATACGCACTCCGTTGCGATTGGTTCACATGGACACACCATCACCGTTAACGCTGCTGGTAACGC
GGAAAACACCGTCAAAAACATCGCATTTAACTATATTGTGAGGCTTGCATAA
```

stfQ **gene (Start and stop codon) (Qin, side tail fiber protein)**

```
GTGAATATAACCGCGCTGACAGACAATACGCAGGGGGCAGCAGGTCTTGAGTTATACGAGGTGTATAACAACGGATA
TCCAACAGCGTATGGAAATATCATTCACCTGAAAGGGATGACAGCCGTTGGCGAAGGTGAGTTACTCATCGGCTGGA
GTGGTACAAGCGGTGCTCATGCTCCGGCATTTATTCGTTCACGACGGGATACGACCGACGCAAACTGGTCGCCGTGG
GCGCAGCTTTACACCTCGGCTCATCCTCCTGCAGAGTTTTATCCAGTCGGTGCACCAATCCCGTGGCCATCAGATAC
CGTTCCGTCTGGTTATGCCCTGATGCAGGGGCAGACTTTTGACAAATCTGCATACCCGAAACTTGCAGTTGCTTATC
                                                               Spacer 12 3'-GTC
CGTCAGGCGTGATCCCTGATATGCGTGGCTGGACGATTAAGGGCAAGCCCGCCAGTGGTCGGGCCGTATTATCTCAG
||||||||||||    || |    ||| ||
GCAGTCCGCACTTTAGAGTGGCAGCAACG-5'
GAACAGGACGGCATTAAATCGCACACCCACAGCGCCAGCGCATCCAGTACGGATTTGGGGACGGAAACCACATCGTC
GTTTGATTACGGAACCAAATCCACGAATAACACCGGGGCGCATACCCATAGTATTAGCGGGACCGCAAATAGTGCCG
GTGCGCACCAACACAAGAGTTCCGGTGCATTTGGTGGCACGAACACGAGCATTTTCCCTAATGGTTATACCGCGATT
TCAAATCTAAGCGCGGGGATTATGAGCACAACAAGCGGTAGTGGCCAGACTCGTAATGCAGGGAAGACATCATCAGA
TGGTGCTCATACCCACTCGCTGTCCGGCACTGCTGCAAGCGCAGGCGCGCATGCACATACTGTCGGTATTGGTGCTC
ATACGCACTCCGTTGCGATTGGTTCACATGGACACACCATCACCGTTAACGCTGCTGGTAACGCGGAAAACACCGTC
AAAAACATCGCATTTAACTATATTGTGAGGCTTGCATAA
```

alpA **gene (Start and stop codon) (CP4-57, transcriptional regulator)**

```
ATGAACAATCGATCGGCCGTTAGAATACTACGGTTACCAGCGGTTATCCAAAAAACAGGTATGGCACGGGCCACCAT
CTATGACTGGTTGAACCCCAAATCACCACGATACGATGCCACCTTTCCCAAAAAGCGAATGCTCGGCGTGAAATCTG
                                                            |  |   ||||||||||||
                                             Spacer 12 3'-GTCGCAGTCCGCACTTTAGAG
TGGCAGCAACG-5'
 |  | |
TCGGATGGATTGAGGCCGAGATTGATGAGTGGTTATCACAACGCTGTAAACTTATTTGA
```

ybcN **gene (Start and stop codon) (DLP-12, DNA base-flipping protein)**

```
ATGCGCACATACAATCCAAACTCTCTTCTCCCTTCACAGATGCAGAAATGCACCTGCAATTCTTTGCATCTAGCGTT
TGACCTCTGCGGAGGGGAAGCGTGAACCTCTCACAAGACGGCATCAAATTACATCGCGGCAACTTCACCGCTATCGG
                   |||||  |  |    |
    Spacer 12 3'-GTCGCAGTCCGCACTTTAGAGTGGCAGCAACG-5'
TCGGCAGATCCAGCCTTATCTGGAGGAGGGCAAATGCTTTCGCATGGTGCTTAAACCGTGGCGTGAGAAACGCAGTC
TTTCCCAGAATGCACTCAGCCACATGTGGTACAGCGAAATCAGTGAATACCTCATCAGCAGGGGTAAAACGTTCGCC
ACTCCAGCTTGGGTAAAAGATGCTCTCAAACACACATATCTCGGTTATGAAACCAAAGACCTGGTTGATGTCGTAAC
CGGTGATATCACCACTATCCAGTCGTTACGCCATACCTCCGATCTTGATACCGGAGAGATGTATGTCTTCCTGTGTA
AGGTTGAAGCCTGGGCGATGAATATTGGTTGCCACCTGACTATTCCACAGAGCTGCGAGTTCCAGCTGCTGCGCGAC
AAGCAGGAGGCGTAATGGCTACACCGCTTATTCGTGTC
```

rzoD **gene (Start and stop codon) (DLP-12, putative prophage lysis lipoprotein)**

```
ATGCGAAAGCTGAAAATGATGCTCTGCGTGATGATGTTGCCGCTGGTCGTCGTCGGTTGCACATCAAAGCAGTCTGT
                                  |  ||      ||    |||||| |
            Spacer 12 3'-GTCGCAGTCCGCACTTTAGAGTGGCAGCAACG-5'                3'-G
CAGTCAGTGCGTGAAGCCACCACGGCCTCCGGCGTGGATAATGCAGCCTCCCCCCGACTGGCAGACACCGCTGAACG
         |||||||  | |   || | |
TCGCAGTCCGCACTTTAGAGTGGCAGCAACG-5' Spacer 12
GGATTATTTCACCCTCAGAGAGAGGCTGA
```

ydfD **gene (Start and stop codon) (Qin, lysis protein)**

```
ATGAATTCAGCATTTGTGCTTGTTCTGACAGTTTTTCTTGTTTCCGGAGAGCCAGTTGATATTGCAGTCAGTGTTCA
                                                                   |||||    | |
                                                Spacer 12 3'-GTCGCAGTCCGCACT
TTAGAGTGGCAGCAACG-5'
 |   ||  |  |  |
CAGGACAATGCAGGAGTGTATGACTGCAGCAACCGAACAGAAAATTCCCGGTAACTGTTACCCGGTCGATAAAGTTA
TTCACCAGGATAATATCGAAATCCCGGCAGGTCTTTAA
    |   |  |     |||||| |  |      |
 3'-GTCGCAGTCCGCACTTTAGAGTGGCAGCAACG-5' Spacer 12
```

tfaP **gene (Start and stop codon) (e14, tail fiber assembly)**

```
ATGAAAATATACTGTTGCTTAAATACCGTTGGTTTTTTTATGGATGGCTGTGGCGTCATTCCGCCAGATTCTAAAGA
AATAACGGCAGAACACTGGCAGTCATTATTAAAATCTCAAGCTGAAGGAGGCGTGATCGATTTTTCTGTTTTTCCTC
CTTCTATTAAAGAGGTTATCCGTACTCATGATGATGAAGTCGCAGATGCGAACTTTCAAAAGCAGATGCTTATCTCT
GATGCAACTGATTTTATCAATAGCAGACAGTGGCAGGGTAAGGCTGCATTGGGAAGACTTAAAGAAGATGAGCTGAA
ACAATATAATTTGTGGCTGGATTATCTGGAAGCACTGGAACTGGTTGATACATCCAGTGCGCCAGATATTGAATGGC
                                  | ||  |    |      |||||||||||||
                   Spacer 13 3'-GTTCACTATAGGTAGTAGCGTAGGTCACGCGG-5'
CTACGCCTCCGGCAGTTCAGGCCAGATGA
```

Supplementary Figure S8. Predicted base-pairing interactions between each of the CRISPR spacers (i.e., spacers 8, 11, 12, and 13) and a protospacer within the cryptic phage mRNA (CPS-53, CP4-6, Rac, Qin, CP4-57, DLP-12, and e14). Note, some spacers match more than one region within a particular cryptic prophage. The complementary nt between the spacers and cryptic prophage protospacers is indicated by yellow highlight and vertical lines. Note that the spacer sequences are written 3' to 5' to aid an understanding of the putative RNAi matching.

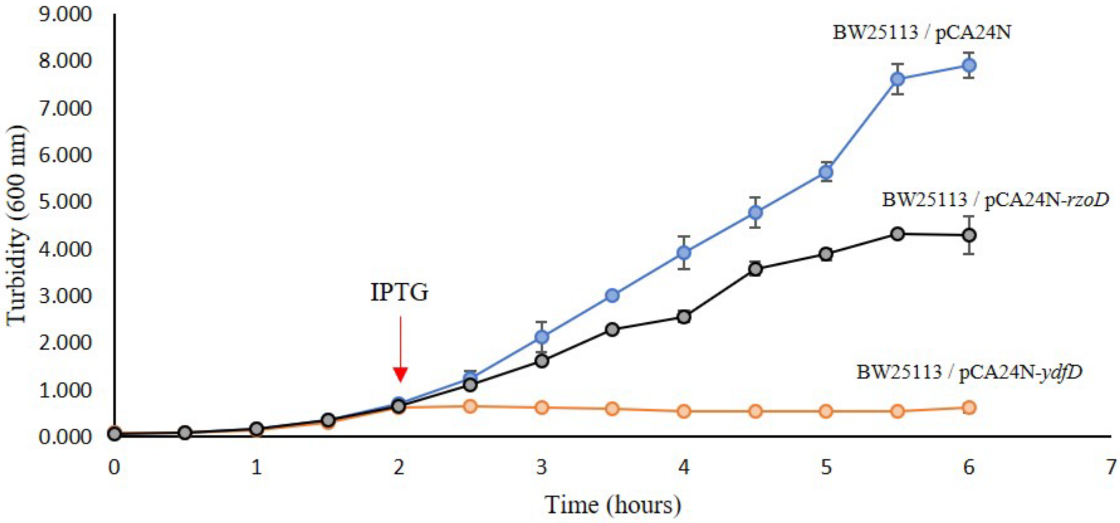

Supplementary Figure S9. Toxicity of YdfD and RzoD. Growth curve at 37°C in LB (turbidity at 600 nm shown) of *E. coli* BW25113 harboring empty plasmid pCA24N (blue line), plasmid pCA24N encoding *ydfD* (orange line), and encoding plasmid pCA24N encoding for *rzoD*. Red arrow indicates addition of 1 mM of IPTG produce the toxins. Average of two independent cultures shown, and error bars indicate standard deviations.

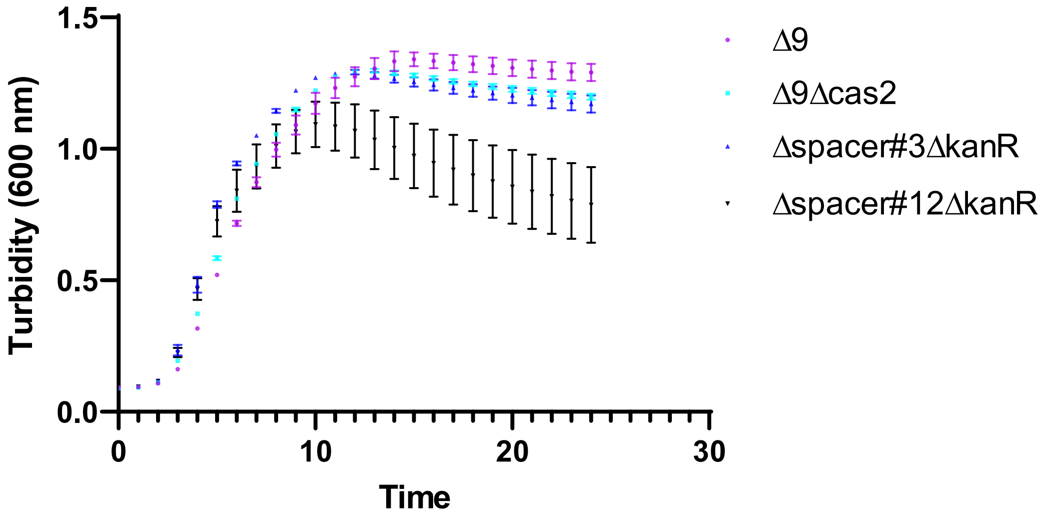

Supplementary Figure S10. Deleting the DNA encoding spacer #12, which encodes crRNA targeting four separate regions in cryptic prophage mRNA (mRNA of Rac, Qin, CP4-57, and DLP-12), leads to lower cell yield. As seen **Table S2** and **Fig. S6**, this is associated with increased cell lysis. In contrast, deleting *cas2* in Δ9 and deleting spacer 3, which encodes crRNA that lacks matches in the mRNA of all 9 cryptic prophages has little effect on growth and cell lysis. Δ9 is BW25113 that lacks all cryptic prophages and Kan^R is kanamycin resistance. Growth (turbidity at 600 nm) at 37°C in LB shown, and error bars indicate standard deviations from two independent cultures.

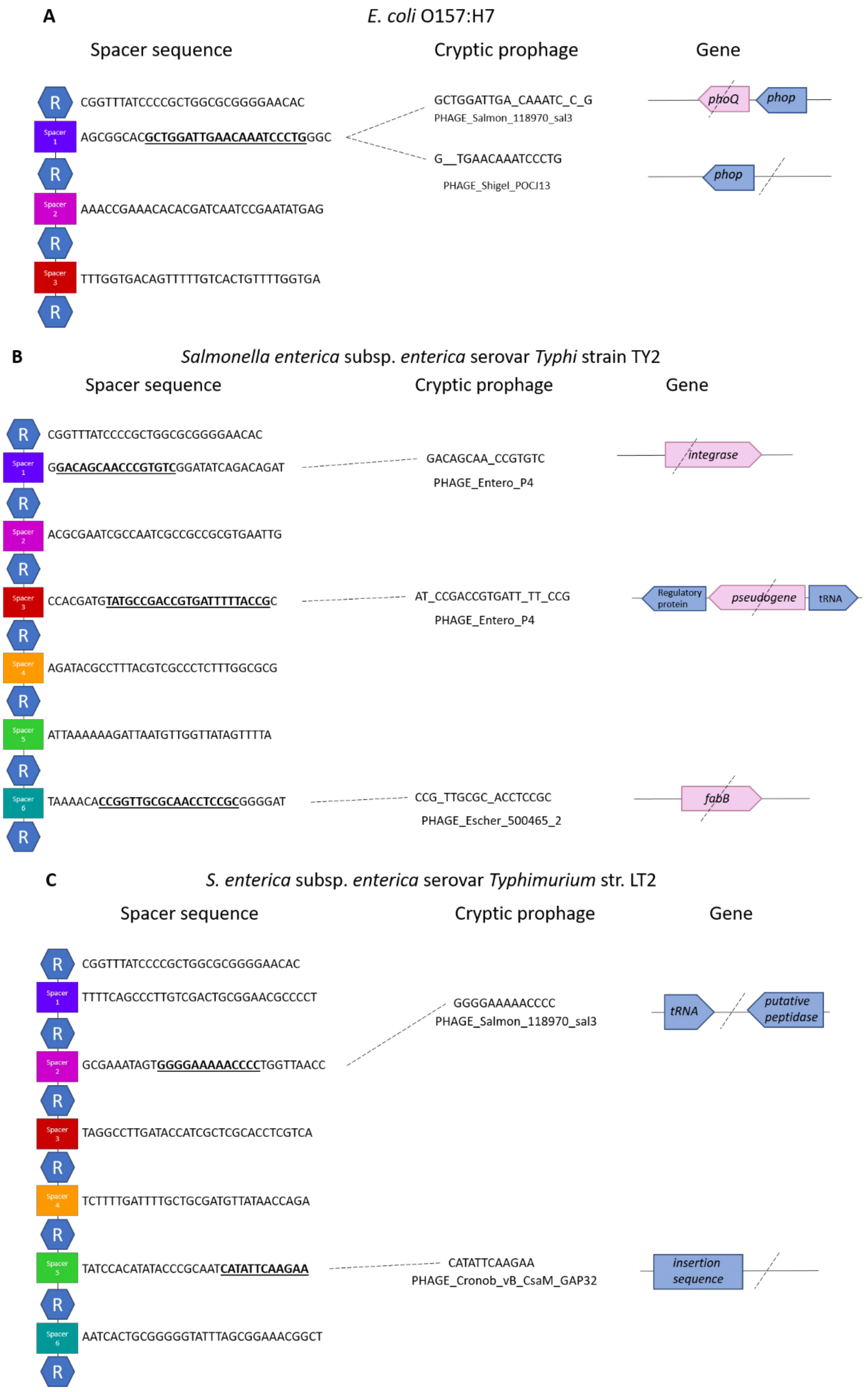
A
E. coli O157:H7
Spacer sequence
Cryptic prophage
Gene
CGGTTTATCCCCGCTGGCGCGGGGAACAC
AGCGGCACGCTGGATTGAACAAATCCCTGGGC
AAACCGAAACACACGATCAATCCGAATATGAG
TTTGGTGACAGTTTTTGTCACTGTTTTGGTGA
GCTGGATTGA_CAAATC_C_G
PHAGE_Salmon_118970_sal3
G__TGAACAAATCCCTG
PHAGE_Shigel_POCJ13
phoQ
phop
phop
B
Salmonella enterica subsp. enterica serovar Typhi strain TY2
Spacer sequence
Cryptic prophage
Gene
CGGTTTATCCCCGCTGGCGCGGGGAACAC
GGACAGCAACCCGTGTCGGATATCAGACAGAT
ACGCGAATCGCCAATCGCCGCCGCGTGAATTG
CCACGATGTATGCCGACCGTGATTTTTACCGC
AGATACGCCTTTACGTCGCCCTCTTTGGCGCG
ATTAAAAAAGATTAATGTTGGTTATAGTTTTA
TAAAACACCGGTTGCGCAACCTCCGCGGGGAT
GACAGCAA_CCGTGTC
PHAGE_Entero_P4
AT_CCGACCGTGATT_TT_CCG
PHAGE_Entero_P4
CCG_TTGCGC_ACCTCCGC
PHAGE_Escher_500465_2
integrase
Regulatory protein
pseudogene
tRNA
fabB
C
S. enterica subsp. enterica serovar Typhimurium str. LT2
Spacer sequence
Cryptic prophage
Gene
CGGTTTATCCCCGCTGGCGCGGGGAACAC
TTTTCAGCCCTTGTCGACTGCGGAACGCCCCT
GCGAAATAGTGGGGAAAAACCCCTGGTTAACC
TAGGCCTTGATACCATCGCTCGCACCTCGTCA
TCTTTTGATTTTGCTGCGATGTTATAACCAGA
TATCCACATATACCCGCAATCATATTCAAGAA
AATCACTGCGGGGGTATTTAGCGGAAACGGCT
GGGGAAAAACCCC
PHAGE_Salmon_118970_sal3
CATATTCAAGAA
PHAGE_Cronob_vB_CsaM_GAP32
tRNA
putative peptidase
insertion sequence

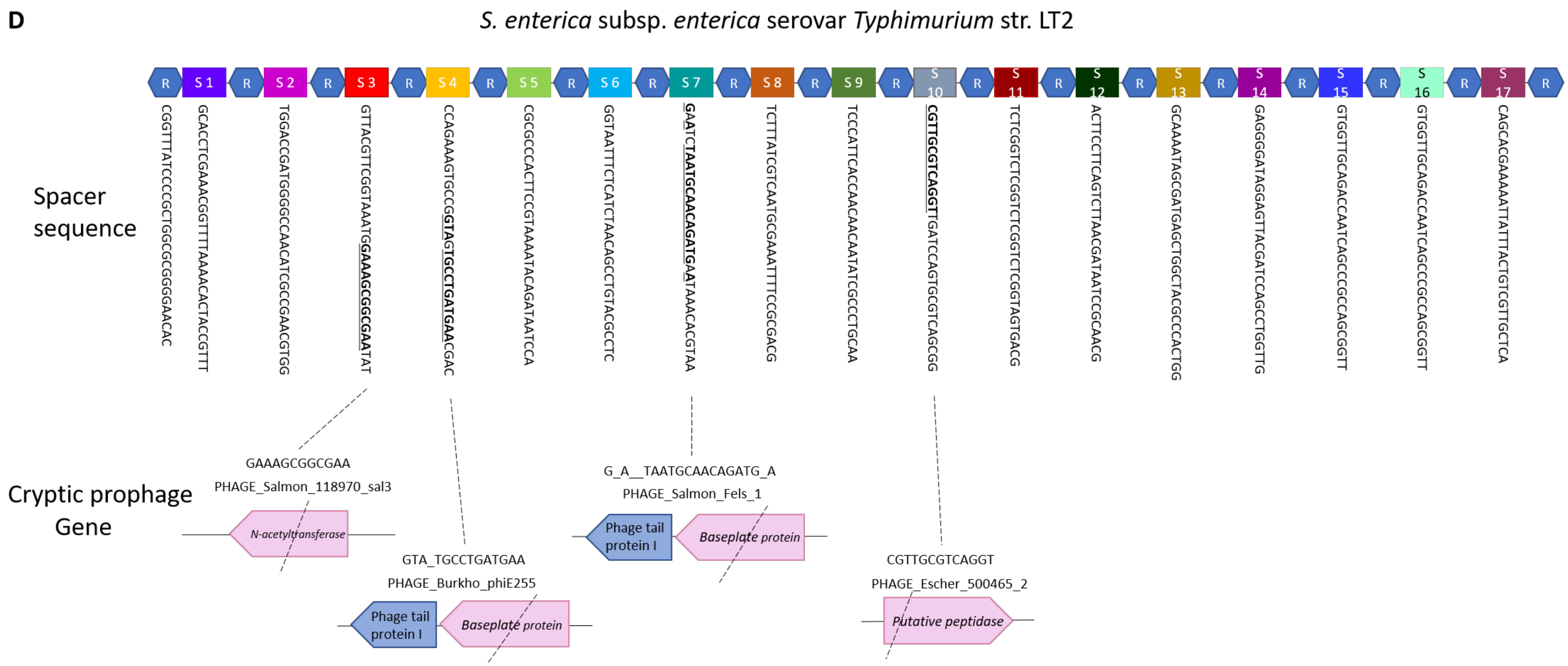
D
S. enterica subsp. enterica serovar Typhimurium str. LT2
R
S 1
S 2
S 3
S 4
S 5
S 6
S 7
S 8
S 9
S 10
S 11
S 12
S 13
S 14
S 15
S 16
S 17
Spacer sequence
CGGTTTATCCCCGCTGGCGCGGGGAACAC
GCACCTCGAAACGGTTTTAAAACACTACCGTTT
TGGACCGATGGGGCCAACATCGCCGAACGTGG
GTTACGTTCGGTAAATGGAAAGCGGCGAATAT
CCAGAAAGTGCCGGTAGTGCCTGATGAACGAC
CGCGCCCACTTCCGTAAAATACAGATAATCCA
GGTAATTTCTCATCTAACAGCCTGTACGCCTC
GAATCTAATGCAACAGATGAATAAACACGTAA
TCTTTATCGTCAATGCGAAATTTTCCGCGACG
TCCCATTCACCAACAACAATATCGCCCTGCAA
CGTTGCGTCAGGTTGATCCAGTGCGTCAGCGG
TCTCGGTCTCGGTCTCGGTCTCGGTAGTGACG
ACTTCCTTCAGTCTTAACGATAATCCGCAACG
GCAAAATAGCGATGAGCTGGCTACGCCCACTGG
GAGGGGATAGGAGTTACGATCCAGCCTGGTTG
GTGGTTGCAGACCAATCAGCCCGCCAGCGGTT
GTGGTTGCAGACCAATCAGCCCGCCAGCGGTT
CAGCACGAAAAATTATTTACTGTCGTTGCTCA
Cryptic prophage Gene
GAAAGCGGCGAA
PHAGE_Salmon_118970_sal3
N-acetyltransferase
GTA_TGCCTGATGAA
PHAGE_Burkho_phiE255
Phage tail protein I
Baseplate protein
G_A__TAATGCAACAGATG_A
PHAGE_Salmon_Fels_1
Phage tail protein I
Baseplate protein
CGTTGCGTCAGGT
PHAGE_Escher_500465_2
Putative peptidase

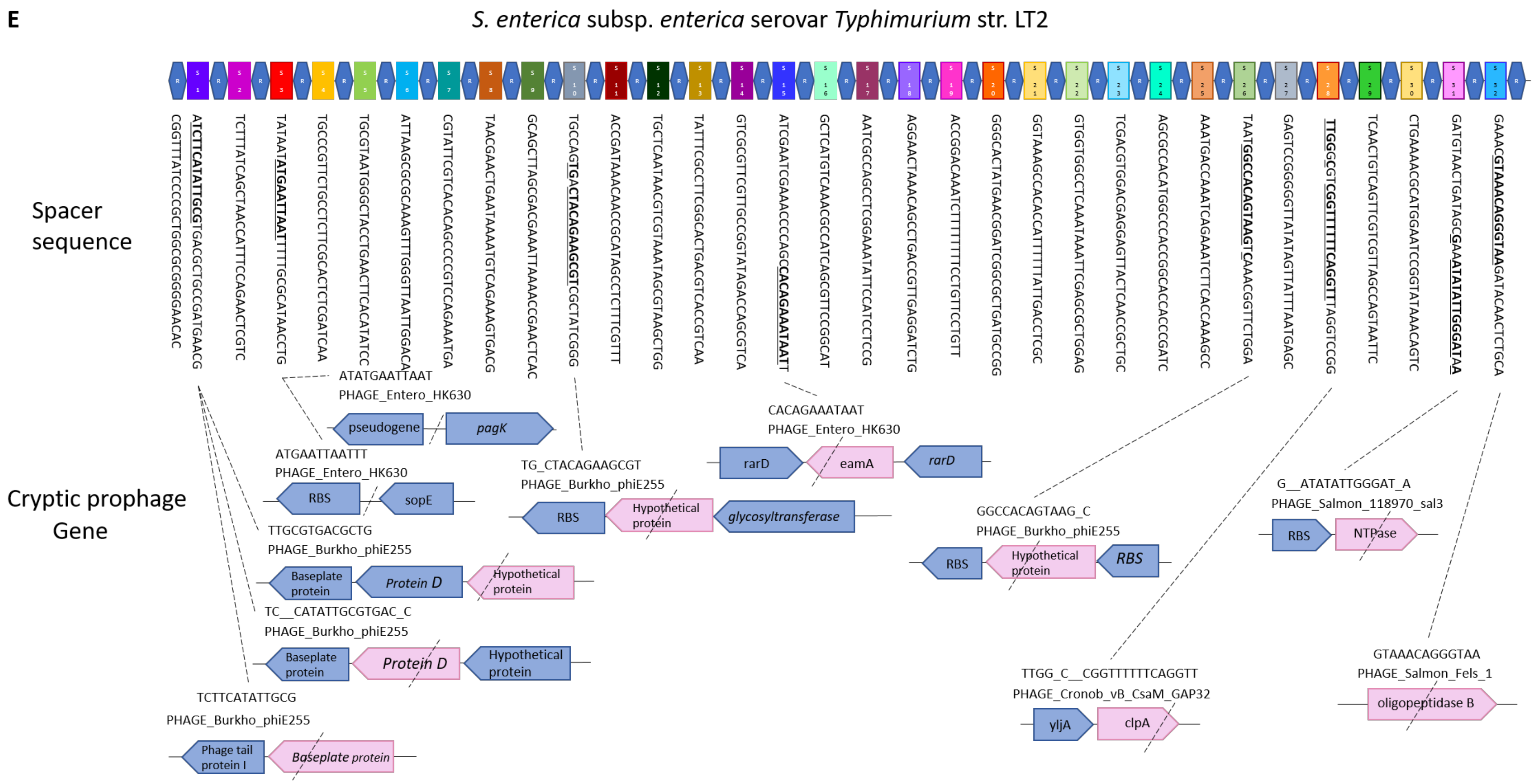
E
S. enterica subsp. enterica serovar Typhimurium str. LT2
Spacer sequence
Cryptic prophage Gene
ATATGAATTAAT
PHAGE_Entero_HK630
pseudogene
pagK
ATGAATTAATTT
PHAGE_Entero_HK630
RBS
sopE
TTGCGTGACGCTG
PHAGE_Burkho_phiE255
Baseplate protein
Protein D
Hypothetical protein
TC__CATATTGCGTGAC_C
PHAGE_Burkho_phiE255
Baseplate protein
Protein D
Hypothetical protein
TCTTCATATTGCG
PHAGE_Burkho_phiE255
Phage tail protein I
Baseplate protein
TG_CTACAGAAGCGT
PHAGE_Burkho_phiE255
RBS
Hypothetical protein
glycosyltransferase
CACAGAAATAAT
PHAGE_Entero_HK630
rarD
eamA
rarD
GGCCACAGTAAG_C
PHAGE_Burkho_phiE255
RBS
Hypothetical protein
RBS
TTGG_C__CGGTTTTTTCAGGTT
PHAGE_Cronob_vB_CsaM_GAP32
yljA
clpA
G__ATATATTGGGAT_A
PHAGE_Salmon_118970_sal3
RBS
NTPase
GTAAACAGGGTAA
PHAGE_Salmon_Fels_1
oligopeptidase B

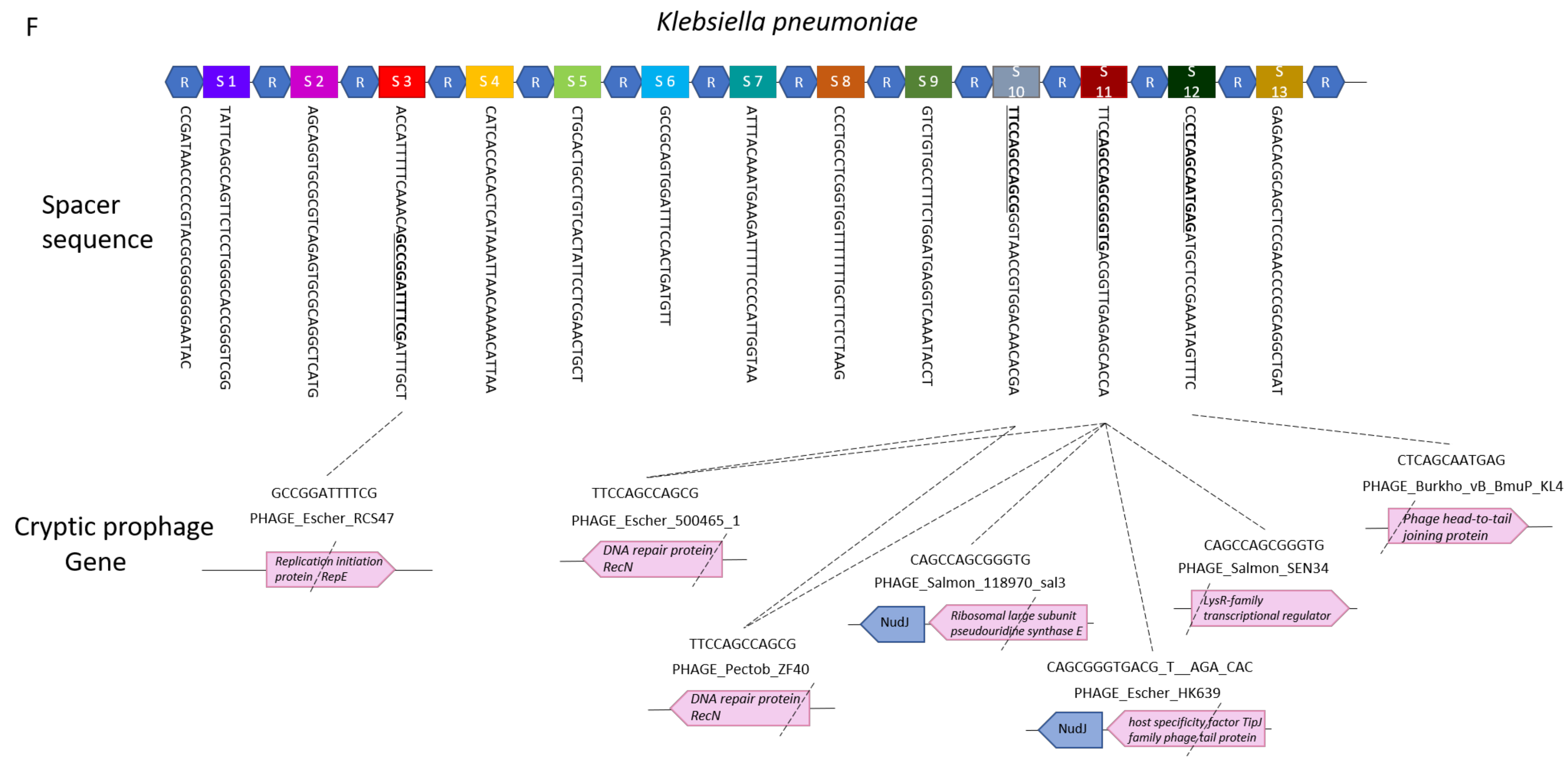

Supplementary Figure S11. ***E. coli* O157:H7 and *Salmonella* spp. CRISPR-Cas spacer sequences related to cryptic prophages. (A)** Repeat (R, blue hexagon) and spacer (rectangle) sequences of each CRISPR-Cas system of *E. coli* O157:H7, *Salmonella* spp., and *K. pneumoniae* indicating matches with their cryptic prophages. (**A**) *E. coli* O157:H7, (**B**) *S. enterica* subsp. *enterica* serovar *Typhi* strain TY2, (**C, D, E**) three CRISPR-Cas systems of *S. enterica* subsp. *enterica* serovar *Typhimurium* str. LT2, and (**F**) *K. pneumoniae*. Pink highlight and dashed lines positions relative to the cryptic prophage genes.
